# Microbial life in preferential flow paths in subsurface clayey till revealed by metataxonomy and metagenomics

**DOI:** 10.1186/s12866-024-03432-z

**Published:** 2024-08-09

**Authors:** Frederik Bak, Christoph Keuschnig, Ole Nybroe, Jens Aamand, Peter R. Jørgensen, Mette H. Nicolaisen, Timothy M. Vogel, Catherine Larose

**Affiliations:** 1 Department of Plant and Environmental Sciences, University of Copenhagen, Frederiksberg, Denmark; 2 Interface Geochemistry, German Research Center for Geosciences, GFZ, Potsdam, Germany; 3 Geological Survey of Denmark and Greenland, Copenhagen, Denmark; 4 M E C ApS, Farum, Denmark; 5 Laboratoire d’Ecologie Microbienne, Universite Claude Bernard Lyon 1, UMR CNRS 5557, UMR INRAE 1418, VetAgro Sup, Villeurbanne, France; 6 IGE – Institut de Géosciences de l’Environnement, Grenoble, France

**Keywords:** Macropores, Shallow subsurface microbiome, Microbial metabolism, Metagenomics, Metagenome-assembled genomes, Biopores, Tectonic fractures

## Abstract

**Background:**

Subsurface microorganisms contribute to important ecosystem services, yet little is known about how the composition of these communities is affected by small scale heterogeneity such as in preferential flow paths including biopores and fractures. This study aimed to provide a more complete characterization of microbial communities from preferential flow paths and matrix sediments of a clayey till to a depth of 400 cm by using 16S rRNA gene and fungal ITS2 amplicon sequencing of environmental DNA. Moreover, shotgun metagenomics was applied to samples from fractures located 150 cm below ground surface (bgs) to investigate the bacterial genomic adaptations resulting from fluctuating exposure to nutrients, oxygen and water.

**Results:**

The microbial communities changed significantly with depth. In addition, the bacterial/archaeal communities in preferential flow paths were significantly different from those in the adjacent matrix sediments, which was not the case for fungal communities. Preferential flow paths contained higher abundances of 16S rRNA and ITS gene copies than the corresponding matrix sediments and more aerobic bacterial taxa than adjacent matrix sediments at 75 and 150 cm bgs. These findings were linked to higher organic carbon and the connectivity of the flow paths to the topsoil as demonstrated by previous dye tracer experiments. Moreover, bacteria, which were differentially more abundant in the fractures than in the matrix sediment at 150 cm bgs, had higher abundances of carbohydrate active enzymes, and a greater potential for mixotrophic growth.

**Conclusions:**

Our results demonstrate that the preferential flow paths in the subsurface are unique niches that are closely connected to water flow and the fluctuating ground water table. Although no difference in fungal communities were observed between these two niches, hydraulically active flow paths contained a significantly higher abundance in fungal, archaeal and bacterial taxa. Metagenomic analysis suggests that bacteria in tectonic fractures have the genetic potential to respond to fluctuating oxygen levels and can degrade organic carbon, which should result in their increased participation in subsurface carbon cycling. This increased microbial abundance and activity needs to be considered in future research and modelling efforts of the soil subsurface.

**Supplementary Information:**

The online version contains supplementary material available at 10.1186/s12866-024-03432-z.

## Background

Soil microorganisms contribute to important ecosystem services including carbon sequestration and nutrient cycling. The effects of both microscale and mesoscale spatial heterogeneity on microbial abundance and community compositions in topsoil have been well studied [1, 2]. However, far less is known about microbial communities and their adaptations to heterogeneities in subsoil. To fully understand the factors shaping subsurface microbial communities, microorganisms in structures contributing to spatial variation in the subsurface such as the preferential flow paths as highlighted by Franklin et al. [3] need to be fully explored.

Clayey tills, heterogeneous glacial sediments deposited during glacial advances, are widely distributed across the Northern Hemisphere [4, 5]. In these areas, subsurface soil contain hydraulic preferential flow paths that can account for > 90% of the water transport, despite their relative volume consisting of less than 1% of the subsoil volume [6, 7]. Below the plough layer, and down to ~ 160 cm below ground surface (bgs), the preferential flow paths are primarily found as earthworm burrows and root channels (biopores), which can be numerous (more than 400 per m^-2^) [8]. Below this depth, the preferential flow paths mainly occur along tectonic fractures, which have been formed during glacial advances 17,000–23,000 years ago [4]. Sediments below ~ 200 cm bgs contain up to 20 stable tectonic fractures per meter [9, 10]. The earthworm burrows and root channels are often interconnected and have been found to be stable and reused by the future generations of worms and roots sometimes over thousands of years [11]. At further depth, paleo-root macropores dating back to ancient indigenous forests can be present in the glacial tills and in some places extending down to 600 cm bgs [11].

While the biopores have been coined microbial hot spots [12, 13], and microbial communities in these niches have been characterized [14, 15], only limited knowledge on bacterial communities is available for fractures [15, 16]. A previous study, covering biopores and tectonic fractures, showed a higher bacterial abundance and diversity in preferential flow paths compared to the adjacent matrix sediments [15]. In addition, taxonomic profiles and abundances of genes involved in nitrogen and sulphur cycling indicated that aerobic conditions expanded to greater depths in the investigated preferential flow paths than in the adjacent matrix sediment. Building upon this knowledge, we aimed to expand our understanding of this subsurface ecosystem by targeting fungal and archaeal communities to provide a more complete representation of subsurface microbial communities. Fungal hyphae are found on soil mineral particles and organic matter and traverse soil pores. They can exploit large soil surface areas for carbon and nutrients and can colonize nutrient rich soil patches [17]. This would suggest the possibility for higher fungal abundance in preferential flow paths.

Preferential flow paths in clayey tills become exposed to water flow following rainfall or irrigation, and, as they remain stable over time, repeated flow occurs within the same pores [18]. Furthermore, the groundwater tables periodically rise, saturating the preferential flow paths with stagnant water, especially during rainy seasons. Since water transport is not constant [3], oxygen and nutrient availability fluctuates over time within the preferential flow paths. As a result, different microbial niches formed by short- and long-term differences in water and nutrient inputs, may exist within clayey tills.

The microorganisms inhabiting the preferential flow paths likely benefit from a nutrient-rich environment derived from plant material descending through these flow paths. However, they might also have to cope with a more variable environment. Consequently, adaptation to varying oxygen levels might be selective and reflected in the bacterial genomic potential. For example, the genome might contain high and low affinity cytochromes or alternative electron acceptors which will allow for oxygen respiration at high to very low oxygen concentrations. The microbial communities and their metabolic potential need to be characterized to understand bacterial lifestyles in preferential flow paths and their influence on nutrient cycling in the subsoil.

In extension of our previous findings for bacterial communities [15], we included archaeal and fungal communities. In addition, using shotgun metagenomics we assembled metagenome-assembled genomes (MAGs) from a hitherto unexplored environment to make inferences of the bacterial genetic potential to adapt to the life in tectonic fractures and the matrix sediment. We hypothesized that the conditions in the preferential flow paths would have significantly higher abundance of fungi and a significant effect on the fungal community. Furthermore, we hypothesized that bacteria found in deep tectonic fractures have adapted to transient input of plant-derived material and fluctuations in oxygen availability. To address these hypotheses, we used amplicon (16S rRNA gene and fungal internal transcribed spacer (ITS) region 2) sequencing to determine microbial community structures in the preferential flow paths and their corresponding matrix sediments in a clay till depth profile down to 400 cm bgs and metagenomics sequencing to identify functional potential.

## Materials and methods

### Site description

**Fig. 1:**
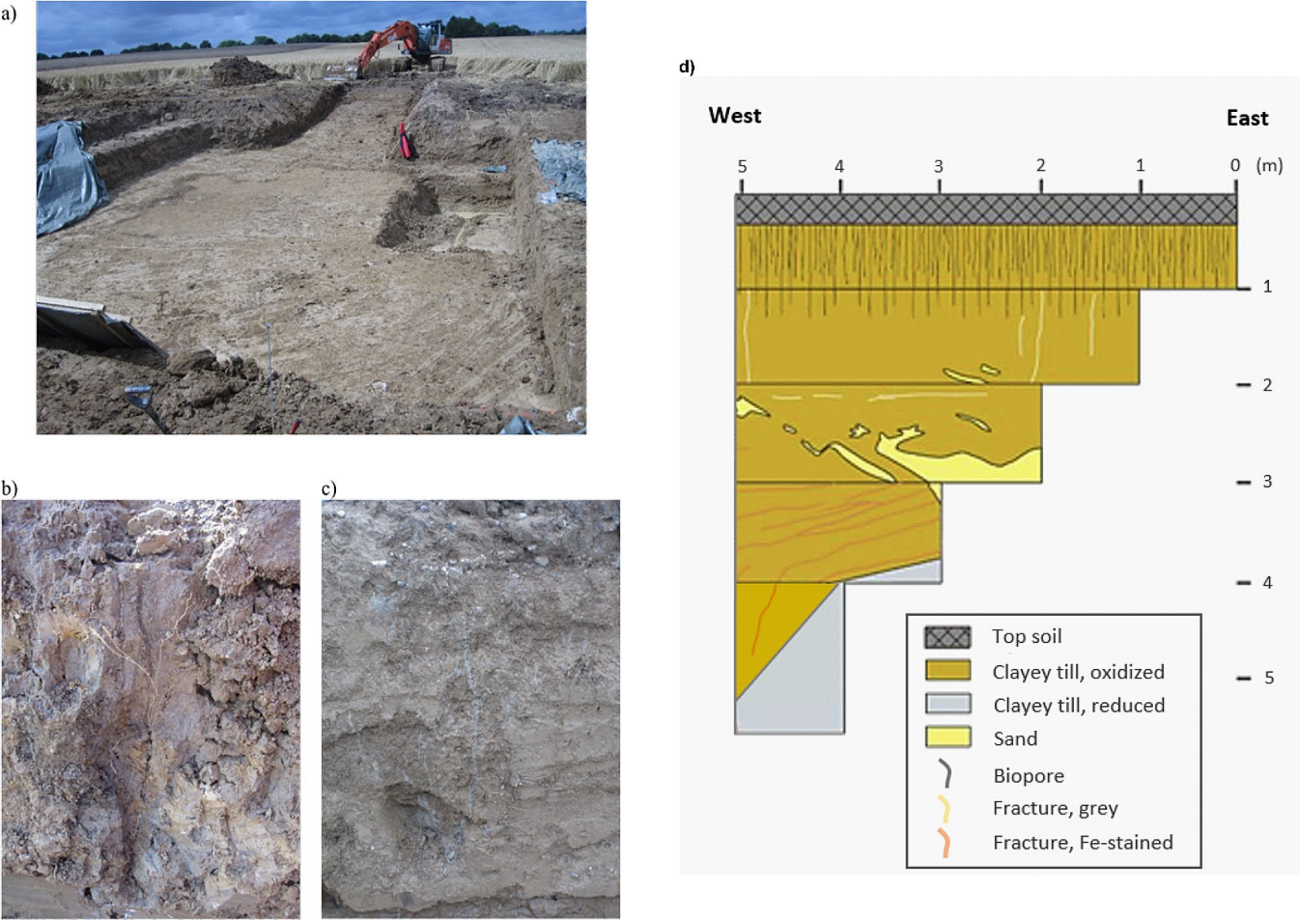
Pictures of the excavation and of selected sampling sites. The site, excavated to a depth of 1 m (**a**). Dimensions are 16 × 16 m horizontally. Pictures of a biopore from 50–100 cm below ground surface (cm bgs) (**b**) and grey fractures100-200 cm bgs (**c**). A schematic drawing of the excavation showing the spatial distribution of biopores and fractures in the excavation (**d**)

The field work of the study was carried out in an excavation (18 × 18 m and 6 m deep) located in a wheat field in Salløv, Denmark (55°33’45.9’’N, 12°06’36.7’’E) (Fig. 1). The fertilizer treatment on the site in the sampling year was nitrogen, phosporous and potassium (NPK) (27-3-5% w/w) with a total of 190 kg N ha^−1^ y^−1^. Pesticides were applied as a mixture of Boxer (Syngenta) and Diflufenican (DFF, Bayer Crop Science). No extra irrigation was performed. The excavation was located on high-ground (43 m above sea level) in an undulating moraine landscape of clayey till deposited during the last glacial maximum approximately 23,000 to 17,000 years BP. The site is in an old agricultural area which has been cultivated since the Danish Bronze Age [19]. Prior to this, it was occupied by dense indigenous forest [11]. The excavation was established for geological studies of fractures and biogenic macropores, and the role of those as preferential flow paths in the till are described in [11] and [20]. The clayey till constituted silty sand and gravely deposits with a clay content of 13–19% and a high content of limestone from 100 cm bgs. Above this depth the limestone content was absent due to intensive chemical weathering. The water table in the till fluctuated between 50 to > 800 cm bgs during the year, and the till matrix had a reddish-brown color to at least 400 cm bgs. The till contained prominent weathered fractures from 50 cm to > 500 cm bgs with mean spacings from a few cm to approximately 30 cm. Below the plough layer (0–30 cm depth) the till was rich in earthworm burrows and root macropores (biopores) to approximately 160 cm bgs. The number of biopores in the matrix sediment reached a maximum of > 500 m^− 2^ at 40–50 cm bgs, dropping to 200 m^− 2^ at 75–100 cm bgs and 40 m^− 2^ at 100–200 cm bgs. From 100 to 300 cm bgs the fractures were bleached to a greyish color (hereafter gray fractures). Deeper fractures had accumulated Fe/Mn-oxides and were reddish stained (hereafter red fractures). Dye tracer hydraulic experiments by Jørgensen et al. (2022) [11] revealed that the worm-burrows constituted major preferential flow paths in the upper 160 cm of the profile. Root macropores continued as dense networks of flow paths located inside the underlying gray fractures (Fig. S1). By the transition to the underlying red fractures, rapid dye tracer flow was halted 3–5 orders of magnitude due to clogging of the fractures with Fe/Mn-oxides (Fig. S1) [11]. This provided a sharp hydraulic barrier with small influence of preferential flow along the deep red tectonic fractures [11].

### Sampling

Soil samples were collected over a 10-day period in August 2017 (see Table S1 for information on sampling dates and precipitation). A 600 cm deep excavation was established to enable sampling from different niches of the till (plough layer, matrix sediments and preferential flow paths) at three sides of the excavation down to a depth of 400 cm bgs (Table 1). Prior to sampling, the outermost part (min. 5 cm) of the excavation sides were removed to avoid any potential contamination. In the plough layer, 12 independent samples were taken (four from each of the three sides of the excavation) with ~ 4 m intervals in between samples. Samples were taken using a sterilized spoon and transferred to Falcon tubes. We collected ~ 2 g of sample for each preferential flow path and 5–10 g for the corresponding matrix. At each depth interval, we collected 12 independent preferential flow path (biopores or fractures) and 12 matrix sediment samples (four from each of the three sides of the excavation). This resulted in a total of 84 samples (Table S1). The depth in the excavation was expanded in one-meter sections and sampling began immediately after each section had been excavated. During night and through periods of rain, a plastic membrane covered the excavation to avoid drying-out or wetting. Each preferential flow path sample represented a unique biopore or fracture, while the corresponding matrix sediment sample was obtained adjacent (2–5 cm) to the preferential flow path and did not contain any material from a preferential flow path. The time between sampling a preferential flow path sample and the corresponding matrix sample was < 5 min, limiting any impact of weather or time. Samples were kept cold (below 10 °C) during transport to the lab for storage at -18 °C until DNA extraction. Samples were not sieved prior to DNA extraction.

A LECO CS-200 induction furnace was used to determine the contents of Total Organic Carbon (TOC, wt%), Total Carbon (TC, wt%), and Total Sulfur (TS, wt%). The samples were crushed to powder and 50 mg was used for TC and TS determination. TOC was determined after removal of carbonate-bonded carbon by HCl. A total of 300 mg was used. The sample was combusted at high temperature and an infrared detector was measuring the produced CO_2_. pH was determined using 0.01 M CaCl_2_.

### DNA extraction and amplicon sequencing

DNA was extracted from the samples using the PowerSoil DNA Isolation kit (Qiagen, Hilden, Germany) as described in [15]. DNA concentrations were measured on a Qubit Fluorimeter (Invitrogen™) and subsequently normalized before library preparation. 16S rRNA gene amplicon libraries targeting the V4 region were prepared following the standard Illumina protocol for amplicon sequencing (“16S metagenomic sequencing library preparation”) using the 515F-Y [21] and 806R [22] primer pairs (Primer sequences are in Table S2). This primer pair was selected as it has been shown to target the highest number of phyla in soil and also targets archaea [23]. To investigate the fungal communities, we targeted the internal transcribed spacer (ITS) region 2, using the primer pair 5.8 S-Fun and ITS4-Fun [24]. All PCR assays were carried out using Platinum Taq polymerase (Invitrogen™). PCR reactions and cycling conditions were performed according to the recommendations in the protocol provided by Illumina. DNA in libraries of single samples was quantified spectrophotometrically and subsequently pooled at equimolar concentration. These final pools were quantified by qPCR using primers annealing to the P5 and P7 flanking regions necessary to perform successful paired-end sequencing and in-house standards (previous successful sequenced libraries). Final pools were also run on an Agilent Bioanalyzer 2100 (DNA 1000) to confirm absence of primer dimers and determine DNA fragment size to calculate molarities. Finally, amplicon libraries were loaded on a V2 flow cell and 2 × 250 bp paired-end sequencing was performed on an Illumina MiSeq platform.

### Analysis of amplicon sequences

For analysis of 16S rRNA gene amplicons, primers were removed from the raw sequences using cutadapt v.1.16 [25]. Quality filtering of raw reads and subsequent sequence analysis was done using the recommendations in the DADA2 v1.8 package [26] in R [27] with the following modifications for 16S rRNA: forward and reverse reads were trimmed to 230 bp and quality filtered (maximum of three expected errors) and merged reads outside the range of 234 and 275 bp were discarded. No clustering of sequences was performed. The resulting amplicon sequence variants (ASVs) were taxonomically classified with the RDP classifier using the Silva v.138 database [28]. The ASVs that were not classified as bacteria or archaea as well as those that belonged to chloroplasts or mitochondria were removed before further analysis. Samples containing less than 1000 reads were filtered, leaving 77 out of 84 samples; Plough Layer 25 cm bgs (*n* = 12), Biopores 75 cm bgs (*n* = 11), Matrix Sediment 75 cm bgs (*n* = 11), Gray Fractures 150 cm bgs (*n* = 10), Matrix Sediment 150 cm bgs (*n* = 12), Red Fractures 350 cm bgs (*n* = 12) and Matrix Sediment 350 cm bgs (*n* = 9). In total, 412,397 reads were obtained from the 77 samples (range 1,182–23,363 reads) after quality filtering. The data comprised 7,139 bacterial and 217 archaeal ASVs. Rarefaction curves confirmed that a sufficient sequencing depth was obtained (Fig. S2).

For ITS sequences, only forward reads were used for further analysis, as some reads did not overlap due the variable length in the ITS region [24]. Primers were removed with cutadapt v.1.16 [25]. Processing was done using dada2 v.1.8 [26]. Quality filtering was performed with maxEE = 1 and truncQ = 11. After chimera removal, taxonomy was assigned using the UNITE database 7.2 [29]. Sequences not classified as fungi and samples containing less than 1000 reads were filtered out, and resulted in 54 total samples: Plough Layer 25 cm bgs (*n* = 12), Preferential Flow Paths 75 cm bgs (*n* = 12), Matrix Sediment 75 cm bgs (*n* = 10), Preferential Flow Paths 150 cm bgs (*n* = 10), Matrix Sediment 150 cm bgs (*n* = 4), Preferential Flow Paths 350 cm bgs (*n* = 2) and Matrix Sediment 350 cm bgs (*n* = 1). In total, 437,070 reads were obtained from 54 samples (range 1006-42,007 reads), comprising 1447 ASVs. Rarefaction curves confirmed a sufficient sequencing depth was obtained (Fig. S3).

### Quantitative PCR

Abundance of the 16S rRNA gene fragments in DNA extracts from the sediment profile was quantified using quantitative PCR (qPCR) in a CFX96 real-time detection system (Bio-Rad Laboratories, Inc., Hercules, CA, USA). The bacterial 16S rRNA gene fragment was amplified using the primer pair 1369F and 1492R and a probe [30] (Table S2). A 10-fold dilution series of *Escherichia coli* K12 DNA was used as standard [15]. PCR amplification was performed in 30 µl reactions containing: 15 µl Lo-ROX Probe Mix (PCR Biosystems, London, UK), 1.2 µl of each primers (100 pmol/µl), 0.6 µl probe (Eurofins Genomics, Galten, Denmark), 11 µl water and 1 µl environmental DNA. The enzyme was activated for 3 min at 95 °C, followed by 40 cycles of 10 s at 95 °C and 30 s at 60 °C. The qPCR was performed on a Biorad CFX Connect Real-Time System. The reaction efficiencies were between 100 and 106%.

PCR amplification of the ITS region 1 was done using the primer pair ITS1-F and ITS2 [31, 32] (Table S2). As a standard, a 10-fold dilutions series of DNA extracted from *Penicillium aculeatum* was prepared, ranging from 10^1^ to 10^7^ copies µl^− 1^. The amplification was performed in 20 µl reactions containing: 10 µl SYBR green Low ROX qPCR Master Mix (Agilent Technologies, Santa Clara, CA, USA), 0.8 µl of both primers (10 µM), 10 µl BSA (20 mg/ml) (Thermo Fisher Scientific, Waltham, MA, USA), 5.4 µl water and 2 µl environmental DNA. After activation of the enzyme for 3 min at 95 °C, amplification was carried out over 40 cycles of 20 s at 95 °C and 30 s at 55 °C. The reaction efficiencies were between 92 and 103%. Six and three samples from the matrix sediments and red fractures at 350 cm bgs were below the detection limit, respectively.

### Analysis of metagenomics reads

Shotgun metagenomic libraries were prepared for samples at 150 cm depth using the same DNA as for qPCR and amplicon sequencing. This depth was selected due to the limited knowledge of microbial communities in tectonic fractures. However, only libraries for six samples were obtained due to low DNA concentrations; three sample from grey fractures and three samples from corresponding matrix sediment. Library preparation and quality check were performed at Eurofins Genomics (Konstanz, Germany) according to their standard protocols. Resulting libraries were sequenced at Eurofins Genomics using Illumina NovaSeq 6000 2 × 150 bp. Raw reads were quality filtered using “iu-filter-quality-minoche” with default parameters in anvi’o v. 7 [33]. This filters read based on B-tail trimming, passed chastity filter, removal of reads which have uncalled bases. Only reads where 2/3 of the bases in the first half of the read have quality Q ≥ 30 are kept [34]. Read statistics can be found in Table S3. All tools were run with default parameters unless otherwise stated. Quality-filtered reads from each niche were co-assembled using MEGAHIT v1.2.9 [35] with a minimum contig length of 1000 bp. Reads were mapped to the contigs using Bowtie2 v.2.4.4 [36] and the recruited reads were stored as BAM files using samtools v.1.13 [37]. Assembled contigs were binned using CONCOCT v.1.1.0 [38].

Bins were manually curated using the interactive interface in anvi’o v.7. Resulting bins were assessed for completeness and contamination/redundancy using CheckM v.1.1.3 [39]. Bins with ≥ 50% completion and less than 10% contamination were classified as medium quality MAGs and used for further analysis (Table S4) [40]. MAGs were dereplicated using drep [41] at 98% ANI. All reads were then mapped back to the dereplicated MAGs using Bowtie2 before reassembly and binning as described above (Table S4). Anvi’o was used to identify open reading frames in the contigs with Prodigal v2.6.3 [42] and using HMMer v.3.3.1 [43] to find single-copy core genes matching the databases of archaeal and bacterial collection. Anvi-run-hmms was used to identify 16S rRNA genes in the MAGs.

We determined the functions of the MAGs using COG20 function, categories and pathways [44], KOfam [45], and KEGG metabolic predictions [46] in the anvi’o workflow. The anvi’o program “anvi-estimate-metabolism” was used to estimate the completeness of the KEGG modules. Genes encoding carbohydrate-degrading enzymes in the CAZymes database were profiled using Diamond [47]. The taxonomy for each MAG was assigned using GTDB-Tk v.1.7.0 [48]. We assessed selected MAG potential to use hydrogen, sulfur or nitrogen as electron donors or acceptors and to aerobically oxidize methane by searching the MAGs for selected enzymes as described in [49, 50] (Table S5). FeGenie was used to annotate genes related to iron oxidation/reduction [51].

### Statistical analysis

The number of 16S rRNA gene or ITS1 copies were not distributed normally (determined by Shapiro-Wilk test at a confidence interval of 95%), and the significance of differences in abundance between preferential flow paths (*n* = 12) and matrix sediments (*n* = 12) at each of the three depths were determined by the Wilcoxon rank-sum test. Community composition was compared using Bray Curtis dissimilarities on raw data and was visualized with non-metric multidimensional scaling (NMDS) ordinations. A permutational multivariate analysis of variance (PERMANOVA) was applied to test for the effect of depth and niche using the adonis2 function with strata in vegan [52]. To test for differences in taxa between niches the Corncob package in R was used [53]. Using 16S rRNA gene V4 reads, the differences in relative abundances of phyla and genera were tested for samples from 75, 150 and 350 cm bgs. For ITS reads, the tests were carried out at the level of phylum and order. *P* values < 0.05 were adjusted for multiple hypothesis testing using the Benjamini Hochberg correction. The cut off for the False Discovery Rate (FDR) was set to q < 0.05. Phyloseq (v.1.28.0) [54], Ampvis2 (v.2.5.1) [55] and ggplot2 (v.3.2.1) [56] were used extensively while working with the data.

## Results

### Preferential flow paths and geochemistry at the site

Below the plough layer, the preferential flow paths in the till profile constituted interconnected surficial worm-burrows and deep root macropores (biopores), and fractures. In the following, those will be referred to as niches in the till distinct from the matrix sediment. The plough layer had the highest concentration of total organic (TOC) and total sulfur (TS) in the profile (Table 1). At 75 cm bgs the biopores contained a substantially higher amount of TOC and total carbon (TC) than the corresponding matrix sediment. This was also the case for the gray fractures at 150 cm bgs although the differences from the matrix sediment were smaller. At 350 cm bgs, the matrix sediment contained higher amounts of TC and TS than the red fractures. pH increased with depth from pH 6.63 in the plough layer to 7.84 at 350 cm bgs.

**Table 1:** Mean sampling depths and the range (in brackets) in depth (cm) below ground surface of the sampled niches (*n* = 12). Mean total organic carbon (TOC), total carbon (TC), total sulphur (TS) and pH of the different niches. pH was not measured (NM) for preferential flow paths due to limited sampling material

Depth [cm bgs]	Niche	TOC (%)	TC (%)	TS (%)	pH (CaCl_2_)
25 (10–30)	Plough Layer	1.08	1.39	0.03	6.63
75 (50–100)	Preferential Flow Paths (Biopores)	0.30	0.44	0.01	NM
75 (50–100)	Matrix Sediment	0.16	0.25	0.01	6.90
150 (100–200)	Preferential Flow Paths (Grey Fractures)	0.12	1.63	0.01	NM
150 (100–200)	Matrix Sediment	0.09	1.38	0.01	7.50
350 (300–400)	Preferential Flow Paths (Red fractures)	0.09	2.79	0.00	NM
350 (300–400)	Matrix Sediment	0.08	3.23	0.01	7.84

### Microbial abundance

**Fig. 2:**
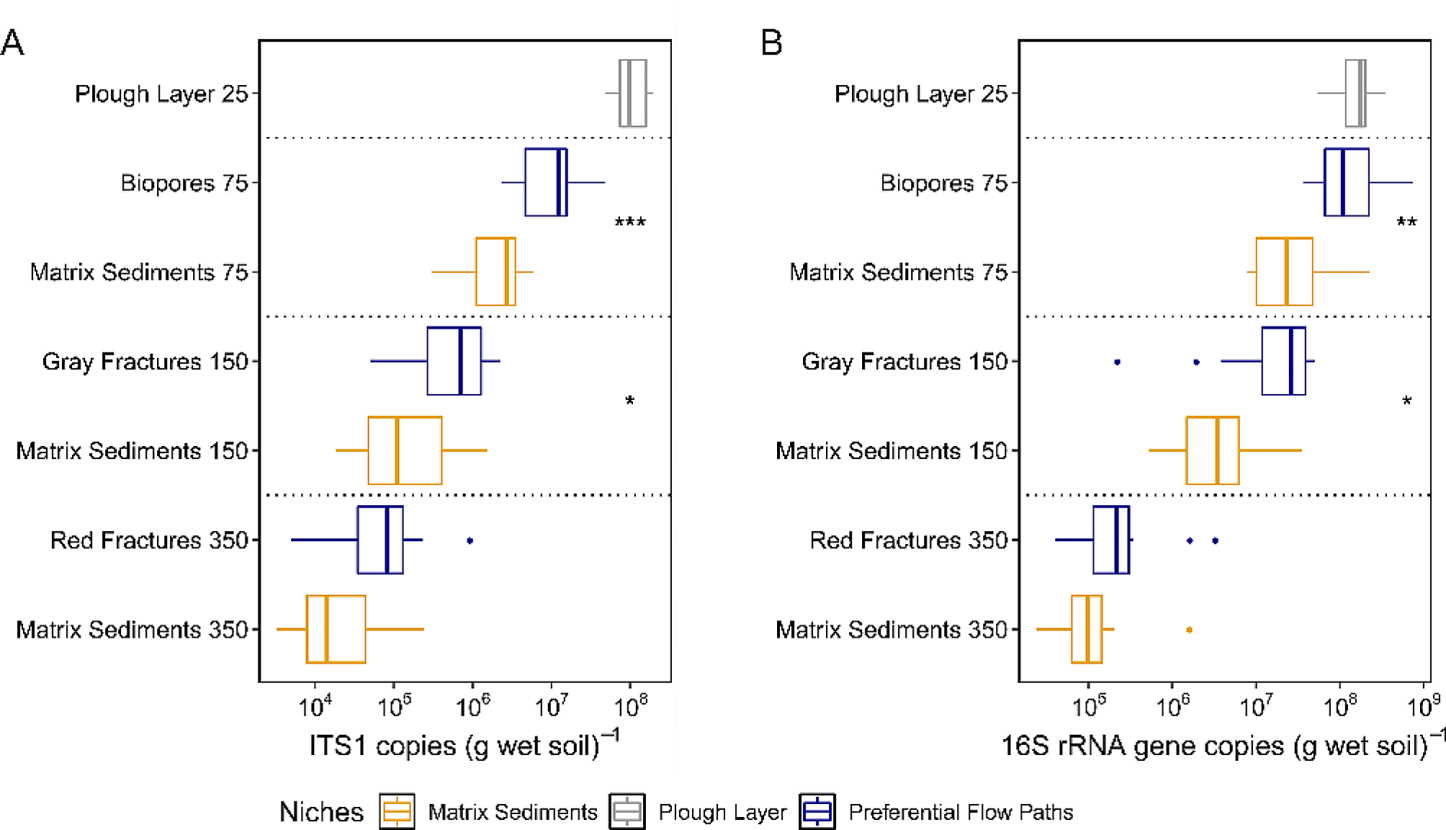
Microbial abundance. Fungal ITS (**A**) and bacterial 16S rRNA gene (**B**) abundance in the clayey till depth profile (16S rRNA gene and ITS1 copy numbers per gram of wet soil, respectively; *n* = 12). Differences were determined by pairwise comparisons using the Wilcoxon rank-sum test and are indicated by asterisks (*, *p* < 0.05, **, *p* < 0.01, ***, *p* < 0.001). The box plots show the median, the two hinges that correspond to the 25th and 75th percentile, and the upper and lower whiskers that extend from higher and lower hinges to the largest and smallest values no further than 1.5 times the inter-quartile range. Points represent outliers. The dashed lines separate the different sampling depths in the profile. Sampling depths (25, 75, 150 and 350 cm depth) are indicated on the y-axis

The number of fungal ITS region 1 copies declined with depth (Fig. 2A). While the mean copy number was 1.13 × 10^8^ copies (g wet soil)^−1^ in the plough layer, it was 10-fold lower in the biopores, and 2,000 fold lower in the matrix sediment at 350 cm bgs. At 75 and 150 cm bgs, the preferential flow paths contained significantly more ITS copies than the matrix sediments (Wilcoxon test, *p* < 0.001 and *p* = 0.024, respectively).

The abundance of bacterial 16S rRNA genes was highest in the plough layer and the biopores at 75 cm bgs (1.79 × 10^8^ and 1.94 × 10^8^ mean 16S rRNA gene copies (g wet soil)^−1^, respectively) (Fig. 2B). The abundance decreased 7-fold from the biopores to the gray fractures at 150 cm bgs and 340-fold to the red fractures at 350 cm bgs. At 75 and 150 cm bgs, the preferential flow paths contained 3.9x and 3.5x more 16S rRNA gene copies than the corresponding matrix sediments (Wilcoxon test, *p* = 0.0011 and *p* = 0.017, respectively), while at 350 cm bgs the difference was not significant (Wilcoxon test, *p* = 0.053).

### Fungal communities

**Fig. 3:**
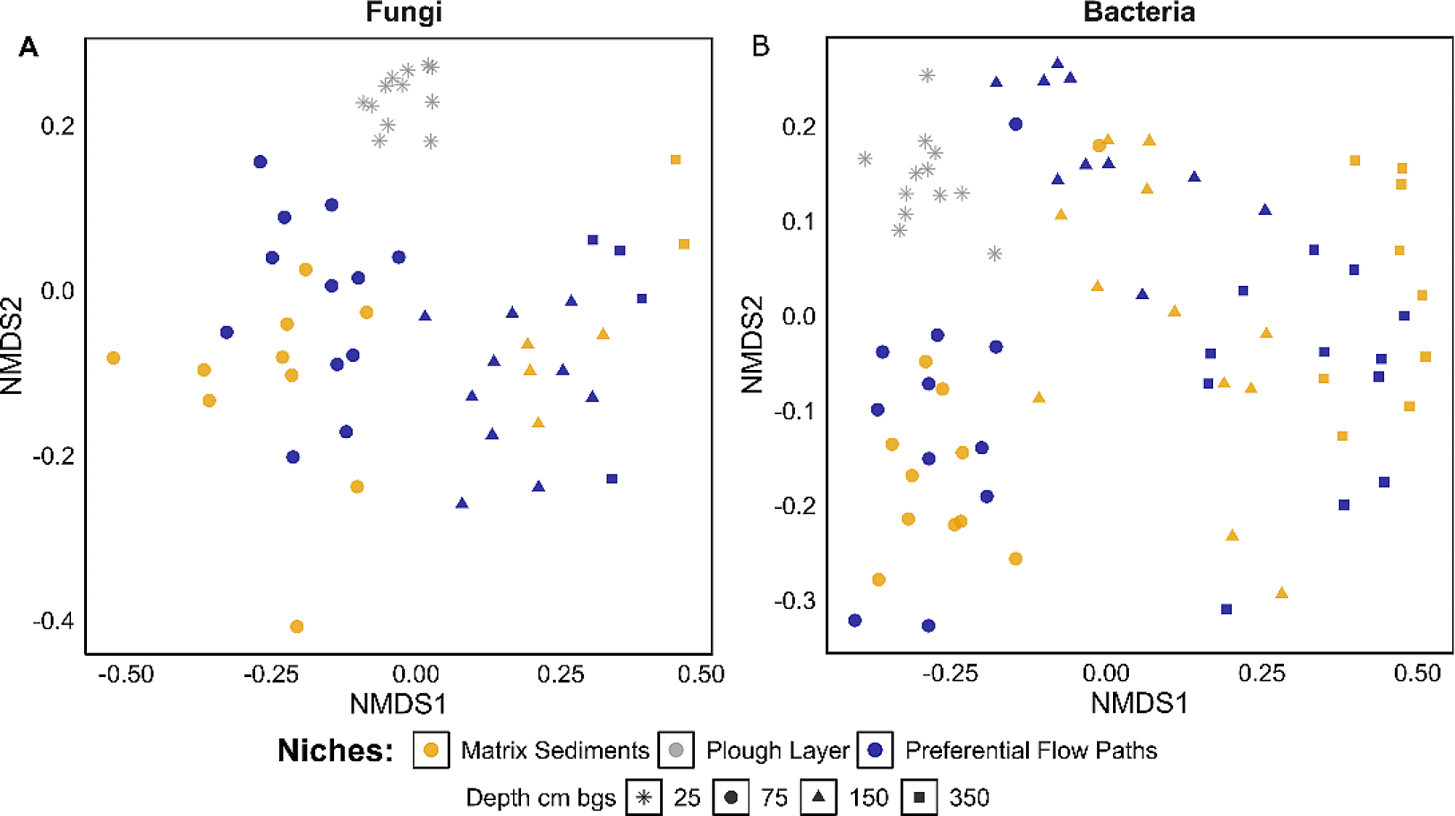
Community compositions. NMDS ordinations of Bray-Curtis dissimilarities between fungal communities (**A**) based on the ITS2 region (stress = 0.18) and bacterial/archaeal communities (**B**) based on the V4 region of the 16S rRNA gene (stress = 0.15). Colors indicate the sampled niches and shapes indicate the sampling depth (cm bgs)

We analyzed the fungal communities in the sediment profile using ITS amplicon sequencing. The variance caused by depth and niche in the community compositions was tested, while the plough layer was excluded as it did not contain preferential flow paths. Depth explained 16% of the variation in fungal community composition (PERMANOVA, *p* < 0.001), whereas niche did not explain any of the variation (*p* = 0.14) (Table S6). The plough layer communities had low variation in community structure between samples (Fig. 3). Below the plough layer, the variation among samples within each niche increased. Ascomycota was the most abundant phylum at all depths, while Basidiomycota and Mortierellamycota were primarily abundant down to 150 cm bgs (Fig. S4). In the plough layer, Hypocreales (Ascomycota) was the most abundant order and decreased with depth as did Filobasidiales (Basidiomycota) and Chaetothyriales (Ascomycota) (Fig. S5). Helotiales (Ascomycota) and Mortierellales (Mortierellomycota) were the most abundant orders at 75 cm bgs but were found at all depths (Fig. S5). Glomerales (Glomeromycota) also peaked at 75 cm bgs but were almost absent from the plough layer (Fig. S5). Xylariales (Ascomycota) increased in abundance with depth and peaked at 150 cm bgs. Moreover, the relative abundance of Pleosporales (Ascomycota), and Capnodiales (Ascomycota) increased with depth. None of the fungal phyla or orders differed significantly in relative abundance between preferential flow paths and the matrix sediments.

### Bacterial and archaeal communities

We analyzed the bacterial and archaeal 16S rRNA gene V4 region amplicon sequences to evaluate community structure. We determined how much of the variance in the bacterial/archaeal community compositions were related by depth and niche, while excluding the plough layer. Depth explained 19% of the variability in community structure below the plough layer (PERMANOVA, *p* < 0.001, Table S7) (Fig. 3). The niche accounted for 2% of the variation for the bacterial/archaeal community compositions in the sampled profile (*p* = 0.018). However, the interaction between the two factors depth and niche accounted for 5% of the variation (R^2^ = 0.05, *p* = 0.001). Hence, we looked at the effect of niche at each individual depth. As samples were taken in pairs, this factor was taken into consideration, since spatial heterogeneity was expected. At all three depths, the niche explained 7–8% of the variation of the community structures (Fig. S6, Table S7). In conclusion, we found that bacterial/archaeal communities colonizing preferential flow paths differ from those inhabiting nearby matrix sediment.

The profile was dominated by bacterial phyla previously found in subsurface soil and their distribution changed with depth (Fig. S7). Verrucomicrobiota and Bacteroidota peaked in relative abundances in the plough layer as well as the biopores and decreased with depth, while the abundances of Chloroflexi, Methylomirabilota and Gemmatimonadota increased with depth. In addition, Myxococcota increased in the deep subsurface at 350 cm bgs, comprising ~ 5% of the community. At 75 cm bgs, Proteobacteria, Myxococcota, Nitrospirota and Latescibacteria were significantly more abundant in the biopores, while the matrix sediments contained higher abundances of Chloroflexi, Actinobacteria and Methylomirabilota (Corncob, FDR adjusted q < 0.05). In the gray fractures at 150 cm bgs, candidate phylum MBNT15 and Verrucomicrobiota were significantly more abundant than in the adjacent matrix, whereas Methylomirabilota and Dadabacteria were more abundant in the matrix sediment (q < 0.05). At 350 cm bgs, the communities in the red fractures had a significantly higher relative abundance of Verrucomicrobiota, Planctomycetota and Zixibacteria than the matrix sediment (q < 0.05), whereas the matrix sediment contained more Chloroflexota, Firmicutes, candidate phyla RCP2-54, Bdellovibrionota and Desulfobacterota.

The plough layer contained 17.4% archaeal sequences, and the biopores and adjacent matrix sediment at 75 cm bgs contained 7.7% and 8.6% archaeal sequences, respectively (Fig. S8). Below this depth, the relative abundance of archaeal sequences increased with depth both in the preferential flow paths and in the matrix sediments, and these sequences comprised 17.9% of the community in the deep matrix sediments at 350 cm bgs. Crenarchaeota dominated in the plough layer while Nanoarchaeota and Thermoplasmatota were almost absent in the top 75 cm of the soil but increased proportionally with increasing depth. The ammonia-oxidizing archaeal families Nitrosospharaceae and Nitrosopumilaceaa comprised the largest proportion of the Crenarchaeota (Fig. S9). The Thaumarcheaota/Cranarchaeota was significantly less abundant in the gray fractures compared to the matrix sediments at 150 cm bgs (q < 0.05).

### Bacterial adaptations to life at 150 cm depth

Bacteria colonizing deep tectonic fractures and adaptation to these interchanging environments are poorly understood. Therefore, we performed shotgun metagenomics on a subset of the samples from 150 cm bgs (Table S4), which was selected due to the limited knowledge of microbial communities in tectonic fractures. Based on the 16S rRNA amplicons, six genera differed significantly between gray fractures and the matrix sediment at 150 cm bgs (Fig. S10). Candidatus Methylomirabilis, and MND1 (Burkholderiales) were enriched in the matrix sediment, whereas IS-44 (Burkholderiales), Candidatus Xiphinematobacter, *Kribbella* and *Rhodoplanes* were enriched in the gray fractures.

**Fig. 4:**
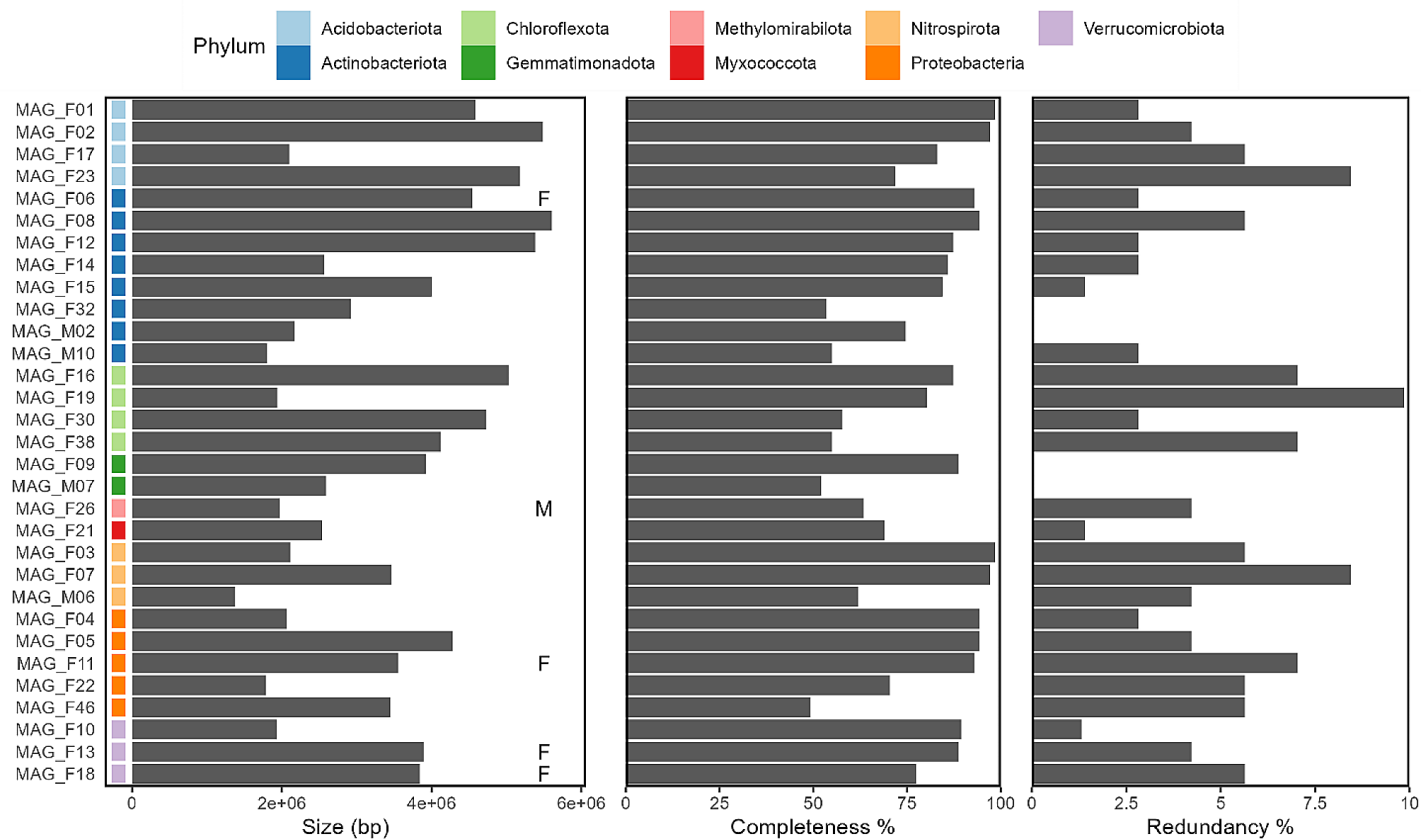
Overview of MAGs. Statistics on their genome size (bp), completeness and redundancy as determined by checkM. Prefixes of MAGs (Fractures and Matrix) refer to the samples used for co-assembly. MAGs that share taxonomy with a genus more abundant in matrix or fractures are annotated with M and F, respectively

We assembled high-quality paired-end metagenome reads from three samples from the matrix sediments and preferential flow paths, respectively (0.3–3.2 mio read pairs per sample) (Table S1, Table S3). Metagenomic binning yielded 31 medium-quality metagenome-assembled genomes (MAGs, < 98% ANI) with > 50% completeness and < 10% redundancy (Table S4; Fig. 4) according to MIMAG standards [40]. Of these, five were > 90% complete and < 5% redundant. Thirty of the MAGs were bacterial, whereas one was archaeal. The MAGs belonged to nine different bacterial and one archaeal phyla: Actinobacteriota (8), Proteobacteria (5), Chloroflexota (4), Acidobacteriota (4), Nitrospirota (3), Gemmatimonadota (2), Verrucomicrobiota (2), Methylomirabilota (1), Myxococcota (1), and Thermoproteota (1). We recovered the (partial) 16S rRNA gene from five of the MAGs (Table S4). Due to high difference in reads between matrix sediment and preferential flow path samples, the highest amount of MAGs (27) were assembled from preferential flow paths.

All the genera which differed in relative abundance (Fig. S10) shared taxonomy with one or more MAGs at the order level or lower (Fig. 4). However, the two genera belonging to the Burkholderiales showed contrasting differential abundance and did not match at the genus level with any of the three Burkholderiales MAGs. Consequently, we restrained from using these MAGs for closer analysis on differences between gray fractures and matrix sediments.

### Predominant anaerobic nitrogen cycling potential in subsurface MAGs

**Fig. 5:**
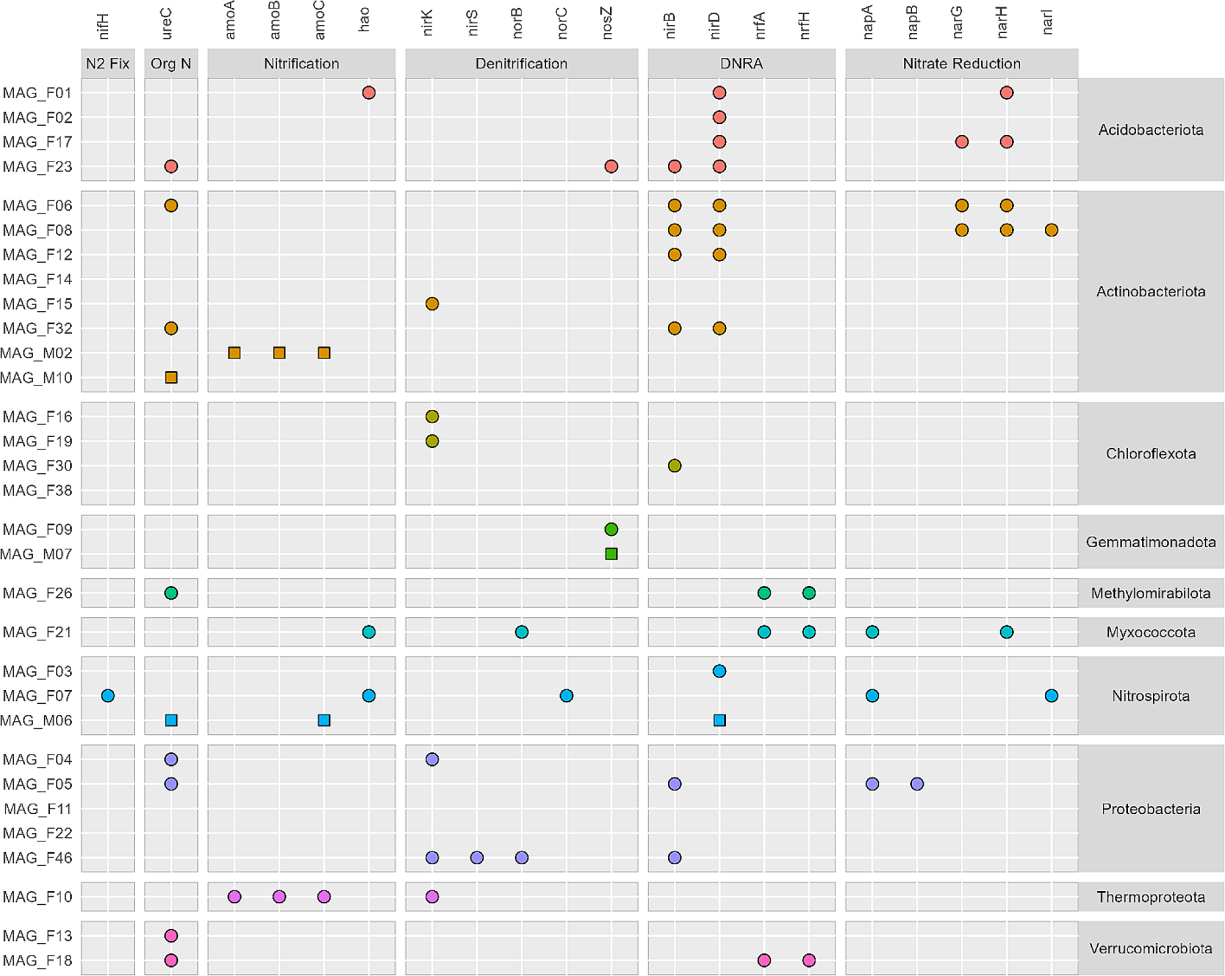
The presence of genes related to N cycling in MAGs from 150 cm bgs. Symbols indicate presence of gene in MAG from gray fractures (circles) or matrix sediment (squares)

A large proportion of the MAGs contained nitrite reductase genes (*nirBD*, *nrfAH*) involved in dissimilarity nitrate reduction to ammonia (DNRA), including MAG_F06 (Actinobacteriota), MAG_F26 (Methylomirabilota), and MAG_F18 (Verrucomicrobiota) (Fig. 5). The indicator genes for denitrification, the nitrite reductases, *nirK* and *nirS* genes, were found in six MAGs. MAG_F46 (Proteobacteria) contained both genes. We found the nitrous oxide reductase gene (*nosZ*) in three MAGs from Gemmatimondadota and Acidobacteriota. Hence, more MAGs showed the potential to DNRA than denitrification. Only two MAGs, MAG_F10 (Thermoproteota) and MAG_M02 (Actinobacteriota) contained the complete ammonia monooxygenase (*amoABC*) operon (Fig. 5) involved in aerobic nitrification. These MAGs lacked hydroxyl amine dehydrogenase (*hao*), while the potential for further oxidation of hydroxyl amine to nitrite was found in in MAG_F01 (Acidobacteriota), MAG_F07 (Nitrospirota) and MAG_F21 (Myxococcota). Besides DNRA, ammonia can be the result of mineralization of organic N such as urea as the *ureC* gene was found in multiple MAGs from several phyla. Nitrogen fixation potential (*nifH*) was only found in MAG_F07 (Nitrospirota). Three MAGs, including MAG_F11 (Proteobacteria), were not found to contain any N cycling genes. We did not find any genes related to anammox nor did have any of the MAGs contain the full comammox pathway.

### Sulfur oxidation potential is common among the subsurface MAGs

**Fig. 6:**
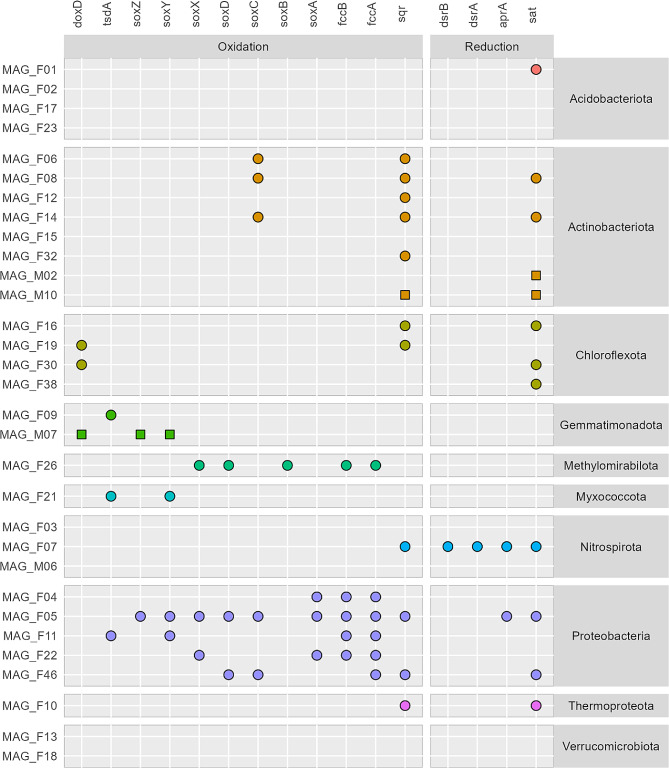
The presence of genes related to S cycling across MAGs from 150 cm bgs. Symbols indicate presence of gene in MAG from gray fractures (circles) or matrix sediment (squares)

The sulfide: quinone oxidoreductases (sqr) used for oxidation of H_2_S were present in multiple MAGs, belonging to the Actinobacteriota, Proteobacteria, Nitrospirota, Thermoproteota and Chloroflexota (Fig. 6). Moreover, MAGs from the Chloroflexota, Gemmatimonadota, Myxoccocota, and Proteobacteria contained the *doxD* or *tsdA* genes involved in the oxidation of thiosulfate. Together with the *fccAB* system found in Proteobacteria, including MAG_F11, and Methylomirabiolota (MAG_F26), and the presence of the SOX system in several MAGs, these findings indicate that oxidation of reduced sulfur is an important metabolism at 150 cm depth. The proteobacteria MAG_F05 contained all SOX genes, except the *soxB* gene. The *sat* gene, which is involved in the initial reduction of sulfate to adenosine-phosphosulphate (APS) was present in MAGs representing multiple phyla. MAG_F07 (Nitrospirota) contained the *dsrAB* genes which catalyze sulfate reduction or function in reverse during sulfide oxidation. Further, this MAG together with MAG_F05 contained the *aprA* gene involved in sulfate reduction. Genes involved in S cycling were almost absent in the four Acidobacteriota MAGs and were not found in Verrucomicrobiota at all.

### Carbon cycling among subsurface MAGs

**Fig. 7:**
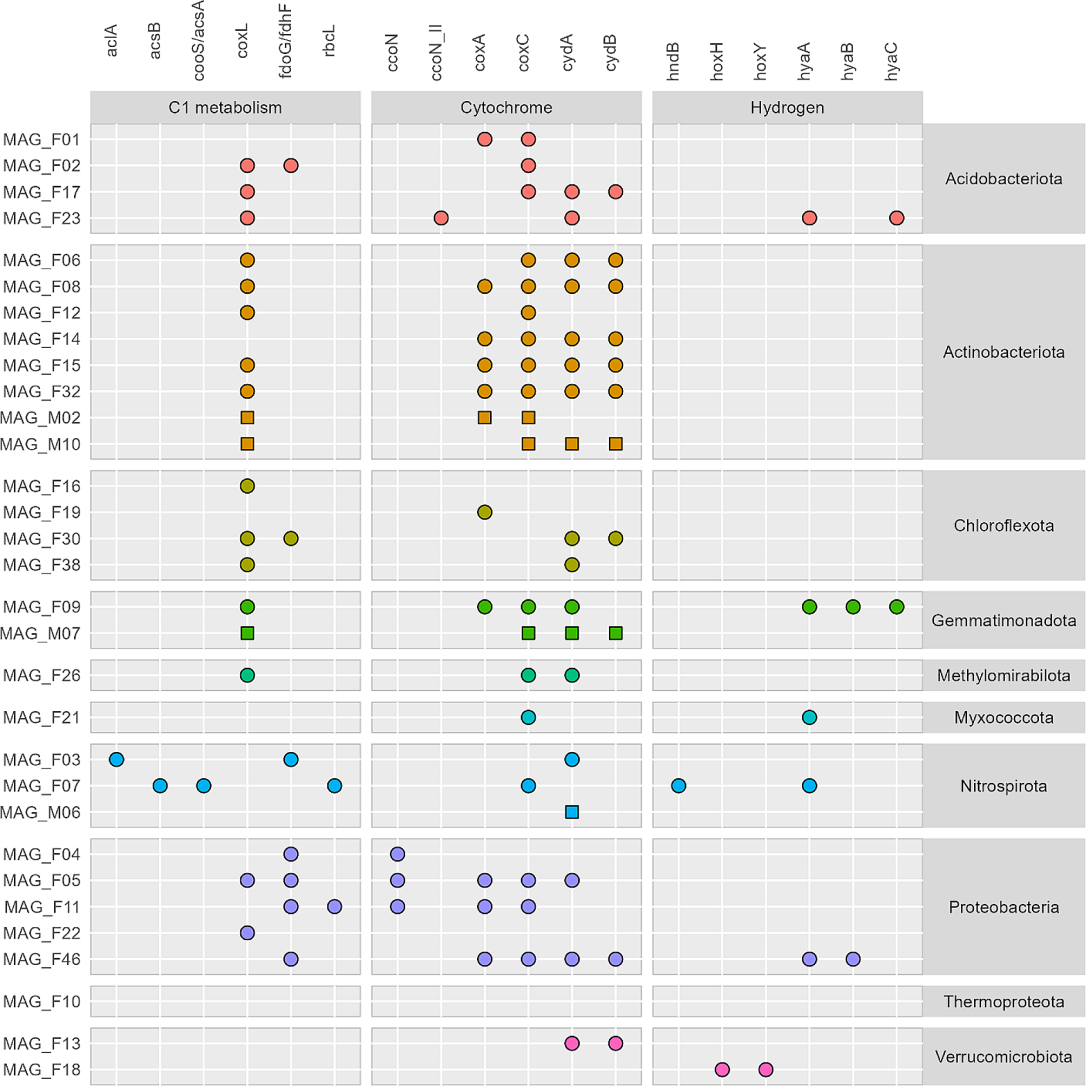
Genes involved in C1 metabolism, cytochromes and hydrogen metabolism in MAGs from 150 cm bgs. Symbols indicate presence of gene in MAG from gray fractures (circles) or matrix sediment (squares)

Carbon monoxide can be an energy and carbon source for soil bacteria. Aerobic oxidation of CO can be performed by bacteria carrying the *coxL* gene, encoding a molybdenum carbon monoxide dehydrogenase (Mo-CODH), which is present in bacteria that can grow chemolithoautotrophically with CO as the energy and carbon source. This gene was found in 18 of the 31 MAGs assembled from the subsoil, belonging to the phyla Acidobacteriota, Actinobacteriota, Chloroflexota, Gemmatimonadota, Methylomirabilota and Proteobacteria (Fig. 7). Seven MAGs from Acidobacteriota, Chloroflexota, Nitrospiriota and Proteobacteria contained the *fdoG* gene encoding a formate dehydrogenase, indicating that they can use formate as an electron donor. Two MAGs, MAG_F10 (Thermoproteota) and MAG_M07 (Gemmatimonadota), contained a *pmoA–*like gene (K10944 – also annotated as *amoA*) indicating potential methane oxidation. None of the MAGs contained the *mcr* gene indicating that none of them were methanogens.

**Fig. 8:**
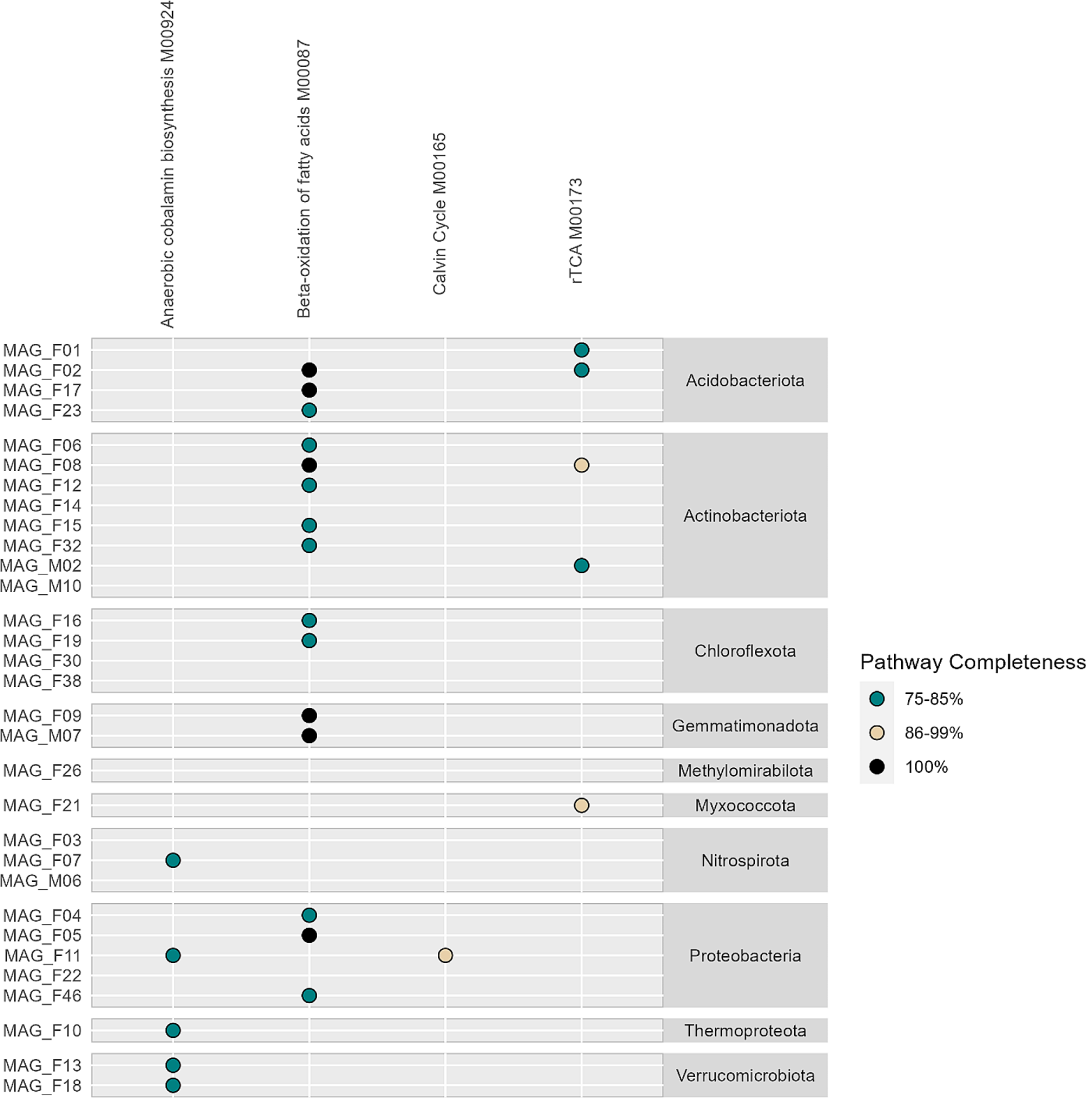
Selected biosynthetic pathways colored by completeness in MAGs. Only pathways which were above 75% complete were included

A few MAGs contained genes indicative of CO_2_ fixation (Fig. 7). Thus, we searched the MAGs for the complete KEGG modules related to the reverse tricaboxylic acid (rTCA) cycle, the Calvin-Benson Cycle, and the Wood-Ljungdahl pathway. MAGs from Acidobacteriota, Actinobacteriota and Myxococcota contained most of the genes in the rTCA cycle (Fig. 8), despite the lack of the *aclA* gene in these MAGs (Fig. 7). In contrast, the *aclA* gene involved in CO_2_ fixation in the rTCA cycle was identified in MAG_F03 (Nitrospirota), but this MAG did not have a near-complete pathway. MAG_F11 (Proteobacteria) had the formate dehydrogenase (*fdoG*) gene as well as the ribulose-1,5-biphosphate carboxylase (*rbcL*) gene, the latter from the CO_2_ fixing Calvin-Benson pathway. This MAG had a near-complete Calvin-Benson pathway (> 90%) (Fig. 8). The Wood-Ljungdahl pathway was not found to be near-complete in any of the MAGs. MAG_F07 (Nitrospirota) was the only MAG that had the gene encoding the anaerobic carbon monoxide dehydrogenase/acetyl-CoA synthase complex (*cooS/acsA*) from this pathway. Finally, the two Verrucomicrobiota MAGs did not contain any genes involved in C1 metabolism.

#### Hydrogen and iron as electron donors

A total of six MAGs contained genes encoding hydrogenases indicating the potential to use hydrogen as electron donor (Fig. 7). These included MAG_F18 (Verrucomicrobiota; *hoxHY* genes of NiFe-hydrogenase groups 3b,3d), MAG_F09 (Gemmatimonadota; *hyaABC* genes encoding NiFe hydrogenase genes in the 1a,1b,1 h or 2a groups), as well as MAG_F21 (Myxococcota), MAG_F23 (Acidobacteriota) and MAG_F46 (Proteobacteria). The latter three MAGs had one or two of the *hyaABC* genes encoding the NiFe hydrogenase. Only one Nitrospirota MAG (MAG_F07) contained *hndB* encoding an NADP + reducing hydrogenase of the FeFe hydrogenase group A.

In addition to the potential use of hydrogen as an electron donor, three MAGs had genes encoding outer membrane cytochromes (Cyc2) for iron oxidation (Table S5). These MAGs belonged to Acidobacteriota (MAG_F01), Proteobacteria (MAG_F11) and Nitrospirota (MAG_F07). MAG_F07 and MAG_F21 (Myxococcota) contained genes (*mtrC*,* dfe*,* omcS*, and *omcF*) involved in iron reduction.

### Subsurface MAGs show potential to thrive at varying oxygen concentrations

The gene encoding for cytochrome *c* oxidase (*coxA*) used for aerobic respiration was found in 11 of the 31 MAGs (Fig. 7). These MAGs were primarily Actinobacteriota or Proteobacteria, while this gene was only found in one MAG from Acidobacteriota, Chloroflexota and Gemmatimonadota, respectively. The gene for aerobic respiration by the low-affinity aa3-cytochrome (*coxC*) was found in 19 MAGs, while MAGs from Verrucomicrobiota, Chloroflexota and Thermoproteota lacked this gene. The microaerophilic cytochrome cbb3 type encoded by the *ccoN* genes was found exclusively in MAGs from Proteobacteria and Acidobacteriota. The cytochrome *bd* oxidase (*cydAB* genes), another high-oxygen affinity terminal oxidase, which is found in microaerophilic organisms was identified in most MAGs across the different phyla. Some MAGs only contained one of the two genes, which could be explained by incomplete genomes. MAGs from Acidobacteriota, Actinobacteriota Gemmatimonadota, Methylomirabiolota and Proteobacteria contained both low- and high-affinity cytochrome oxidases allowing them to thrive under a range of oxygen concentrations. Only one of the Verrucomicrobiota MAGs (MAG_F13) contained genes encoding for a cytochrome oxidase.

### Broad repertoire of carbohydrate active enzymes among subsurface MAGs

Considering the possible downwards transport of organic carbon, we searched the MAGs for carbohydrate active enzymes (CAZymes). MAG_F06 (Actinobacteriota) had more than 400 enzymes related to degradation of carbohydrates and was the one containing most of these enzymes (Fig. S11). Four other MAGs from Acidobacteriota, Chloroflexota and Proteobacteria contained more than 200 CAZymes. Especially enzymes involved in the degradation of xylan, peptidoglycan, chitin, and beta glucan were found in high numbers in these MAGs. MAGs from the Nitrospirota, Gemmatimonadota and Thermoproteaota had the lowest number of CAZymes. Notable differences in number of CAZymes for MAGs were observed within Acidobacteriota, Actinobacteriota and Chloroflexota, and could not be linked to differences in completeness of these MAGs. In contrast, MAGs within Nitrospirota and Verrucomicrobiota had similar distribution of CAZymes, respectively. The proportion of CAZymes within each MAG was comparable across the different phyla, with few notable variations, exemplified by the high proportion of xylanases in MAG-F10 (Thermoproteota) and very low proportion of enzymes degrading peptidoglycan in Verrucomicrobiota. For the MAGs belonging to the latter phyla, higher proportion of enzymes related to degradation of mannan and beta glucan were found. Plants contain multiple fatty acids that can serve as carbon sources for bacteria. Hence, we complemented our search of CAZymes by looking for metabolism potential in the MAGs. We found six MAGs that contained the full pathway for beta-oxidation of fatty acids, belonging to Acidobacteriota, Actinobacteriota, Gemmatimonadota and Proteobacteria (Fig. 8). In addition, near complete pathways (> 75%) were found in nine MAGs from these phyla including Chloroflexota.

### Anaerobic cobalamin biosynthesis potential dominates in subsurface MAGs

We searched the complete KEGG modules for the ability to synthesize cobalamin (vitamin B12), an important precursor for multiple reactions. The molecule can be synthesized both anaerobically and aerobically. None of the MAGs contained a complete or near complete (> 75%) aerobic pathway. In contrast, five MAGs, the Nitrospirota MAG_F07, Rhizobiales MAG_F11, Thermoproteota MAG_F10 and the two verrucmicrobial MAGs, MAG_F13 and MAG_F18 had near complete anaerobic pathways (Fig. 8). Moreover, we found one or more genes encoding cobalamin-dependent enzymes (methylmalonyl-CoA mutase, *mutA*, methionine synthase, *metH* and ribosomal RNA small subunit methyltransferase, *rsmB*) in 28 of the 31 MAGs (Table S5). The missing genes in three MAGs could be explained by lower completeness in these genomes, especially since MAG_F38 from the Anaerolineae is from the same order as MAG16 that contained two of the searched genes.

## Discussion

Here, we characterized the microbial communities in preferential flow paths and adjacent matrix sediments at different depths to 350 cm bgs and used shotgun metagenomics to focus on the physiology of MAGs representing bacteria colonizing gray fractures at 150 cm bgs. To our knowledge, this is the first characterization of fungal communities in deep preferential flow paths.

### Microbial communities are shaped by depth

The 16S rRNA genes and ITS copies declined with depth as shown previously [15, 57, 58], indicating a decrease in microbial abundance. Soil depth shaped the fungal community composition down to 350 cm bgs in the matrix and the preferential flow paths. For the soil matrix, this is consistent with previous studies of wheat fields [59] or forest soil down to 100 cm bgs [60]. We found that saprotrophic fungi such as Hypocreales, Helotiales, and Mortierellales (FUNGuild classification [61]) dominated shallower depths (25 and 75 cm bgs), and decreased with depth. This probably reflects the decrease of plant roots, root exudates as well as available dead organic matter with depth. Below this zone, Xylariales and Capnodiales, increased in relative abundance. Both orders include plant pathogens as well as members grouped among the black fungi, which represent a heterogeneous taxonomic group that includes the most stress-resistant eukaryotes known to date [62].

The low relative abundance of Glomerales in the plough layer, might reflect a negative impact by tillage [63]. As they form arbuscular symbioses with plants [64], they might establish interactions with deep parts of the root systems of the wheat grown on the field, explaining their higher abundance below the plough layer. Actively growing roots or decomposed organic material may create patches of nutrients in both preferential flow paths and matrix sediment, which can explain the increasing variation of fungal communities with depth, as has been shown by Schlatter et al. (2018) [59] for the upper 100 cm of the soil profile.

The composition of the bacterial and archaeal communities varied vertically in the matrix sediment as seen from other soil types, and for the preferential flow paths as well [15, 65, 66]. Multiple phyla showed similar trends in relative abundance when compared with the results from Bak et al. (2019) [15]. For example, the Acidobacteriota was stable in relative abundance in the depth profile, Chloroflexi increased with depth, and Verrucomicrobiota decreased with depth. The decrease in relative abundance of Nitrososphaeraceae between 150 cm and 350 cm indicates a shift in ammonia availability, as members of this family have low ammonia affinity [67], although changes in oxygen conditions or other environmental conditions could play a role as well. Supporting this, Wu et al. (2023) [66] found a substantial decrease in ammonia oxidation potential below 300 cm.

### Preferential flow paths shape bacterial and archaeal communities, not fungal

The higher gene copy numbers in the preferential flow paths at 75 and 150 cm bgs are in line with previous findings for bacteria [15]. This pattern coincided with the availability of TOC. High nutrient concentration in biopores turns these niches into hot spots [12], suggesting the gray fractures at 150 cm bgs are subsurface hot spots as well. At this site, TOC content may be linked to water flow, as hydraulic experiments showed multiple orders of magnitude less preferential water flow in the red fractures [11], resulting in less TOC and thereby microbial abundance.

Interestingly, and contrasting our hypothesis, fungal community compositions were not affected by the preferential flow paths. Soil fungi create long mycelial network [68]. Given the short distances between biopore or fracture samples from surrounding matrix (< 5 cm), this may explain the lack of differences between communities. As root-colonizing fungi can translocate nutrients through overlapping mycelia [69, 70], it suggests that fungi transport nutrients from the preferential flow paths into the matrix sediments.

The composition of bacterial and archaeal communities was affected by the presence of flow paths at all depths, consolidating observations reported by [15]. Bak et al. (2019) [15] found Verrucomicrobiota and Nitrospirota in higher relative abundance in biopores and in fractures below 300 cm bgs compared to matrix sediments, respectively. Together, this suggest that phyla such as Verrucomicrobiota and Nitrospirota are better adapted to the conditions in preferential flow paths, but that site specific conditions determine at what depth they are most dominant.

Taken together, the community compositions and abundances point towards the influx of organic carbon as an important factor in shaping the subsurface bacterial and archaeal communities, as discussed above, although oxygen and nitrogen play a role as well (discussed more detailed below).

### Bacterial adaptations at 150 cm bgs

The fractures appearing from 100 to 300 cm bgs were grayish. This reflects the temporary water saturation and degradation of vegetative organic matter (e.g. decaying roots along anaerobic micro-sites) at the site, resulting in reduction and removal of Fe/Mn-oxides with the water flow [71–73]. At 150 cm bgs, four genera were found to be significantly enriched in the gray fractures according to the 16S rRNA amplicon data. Putatively assigning these enriched genera to MAGs could reveal traits explaining their differential abundances between gray fractures and the matrix sediment. Consequently, we highlight unique functions that would enhance their capacity for survival.

A higher prevalence of genes related to DNRA compared to denitrification genes indicate that DNRA is favored in the gray fractures. For example, *Kribbella* (MAG_F06) possessed the ability to dissimilatory reduction of nitrate and was enriched in the gray fractures. The ability to reduce nitrate has been shown for several *Kribbella* strains [74]. An increased amount of labile C increases the respiration and consequently a shift from denitrification to DNRA [75]. However, DNRA rates depend on soil type, and both DNRA and denitrification rates increases with soil moisture [75]. Still, the input of organic C from above in the soil profile might cause the higher prevalence of DNRA genes in the MAGs.

The finding that only three MAGs contained the *nosZ* gene, further supports the notion of denitrification is less dominant than DNRA in the gray fractures. The presence of this gene in Gemmatimonadota MAG_F09 from the Gemmatimonadales order corroborates findings of a *Gemmatimonas* sp., which reduces nitrous oxide only after O_2_ exposure and sustains viability during anoxic periods although without growing [76]. In the 16S rRNA amplicon data, Gemmatimonadaceae within the order Gemmatimonadales, comprised ~ 5% of the communities at 150 cm. The ability to switch to N_2_O reduction during periods with low oxygen concentrations can be advantageous in an environment with fluctuating oxygen concentrations, corroborating the seasonal water table fluctuations from 100 to 600 cm bgs [11]. As the relationship between DNRA and denitrification rates impacts N_2_O release from soil, the rates of these processes warrant further research, especially seen in the light of the high number of fractures found in the subsurface.

Both DNRA and denitrification might occur at water saturation either after rainfall or when rising ground water table creates temporarily microaerophilic or anoxic conditions in the subsurface, as seen in the saturated zone [66] and upon rewetting in topsoil [75].

Nitrogen fixation potential was solely found in one MAG from 150 cm bgs, and thus, it appears not to be an important source for ammonia at this depth. Instead, the widespread occurrence of urease genes across multiple phyla suggests the cycling of organic N potentially originating from wheat roots found in the gray fractures at this depth or transported from above. For example, the Candidatus Xiphinematobacter from Chtoniobacterales (sharing taxonomy with MAG_F13 and MAG_F18 at the order level), which was enriched in the gray fractures, has the potential to hydrolyze urea to access N originating from organic material and generate ammonia. Indeed, the gray fractures at 150 cm depth harbored high relative abundances of known ammonia oxidizing archaea and bacteria (Nitrosophaeraceae, Nitrosopumilaceae and Nitrosomonadaceae) in agreement with the findings in Bak et al. (2019) for fractures at 230 cm bgs. The finding indicates available ammonia at this depth, and the *amoA* gene was further detected in Thermoproteota and Actinobacteriota MAGs. Hence, despite the predominant anaerobic N cycling processes, aerobic N cycling takes place. These findings are consistent with the hydrological observations from the site [11], suggesting that the fluctuating groundwater table plays a key role for microbial nutrient turnover.

Multiple MAGs harbored the potential to obtain energy from sulfide oxidation, including the enriched genus *Kribbella* (MAG_F06). The potential to oxidize H_2_S and CO found in this MAG indicates a facultative chemolitotrophic lifestyle, although it calls for experimental validation. The genus is primarily considered chemoorganotrophic [77], but there are indications of increased abundance of *Kribbella* upon emission of H_2_ in the rhizosphere of soybean [78], suggesting a chemolithotroph lifestyle.

In the gray fractures, sulfate reduction may not be the source of sulfide, as only one MAG (MAG_F07) contained the *dsrAB* together with the *aprA* and *sat* genes, suggesting it’s potential in sulfate reduction [79]. Bak et al. (2019) did not detect any *dsrB* genes in the top 300 cm of the sediment profile, indicating that produced sulfide should diffuse more than 100 cm in the sediment profile. This renders sulfate reduction unlikely as a source of H_2_S, as also proposed for a marine environment [80]. Instead, we speculate that microorganisms metabolize sulfur containing amino acids from plants, fungi or bacteria, as these are among the most common sulfur organic compounds in soil [81]. This metabolism may lead to a release of H_2_S, which in turn can be oxidized to sulfite or further to sulfate and be taken up by plants or for microorganisms.

Although hydrogenases were found in MAGs spanning multiple phyla, only six MAGs contained hydrogenases suggesting that hydrogen as an electron donor is less important than sulfide oxidation. It can be used as an alternative energy source when other electron donors are scarce and allow bacteria to go into dormancy until more favorable conditions occur [82]. Hydrogen is commonly available in the topsoil because of nitrogen fixation, fermentation or acetate oxidation. At this site, only one nitrogen fixing bacteria (MAG_F07) was identified, indicating that nitrogen fixation is a limited source of hydrogen at this depth.

The higher prevalence of aerobic compared to anaerobic CO dehydrogenases (*coxL* genes) in the subsurface MAGs supports the notion of this part of the subsurface being aerobic, at least periodically, as discussed above. The potential for CO oxidation was present in *Kribbella*, which was enriched in the gray fractures. CO is emitted from plant roots [83] and the potential for CO oxidation widespread among soil taxa [84], consistent with our findings. Further, Cordero et al. (2019) showed that *Mycobacterium smegmatis* increased transcription of CO dehydrogenase 50-fold after organic carbon limitation, allowing the strain to survive but not grow [84]. We speculate that other soil bacteria can respire CO to survive during organic carbon starvation; a phenomenon which may transiently occur in the gray fractures, although more research is needed to confirm this. The presence of low- and high-affinity cytochrome oxidases found in multiple MAGs provides the genomic potential to thrive at atmospheric and microoxic conditions. This is common in Acidobacteriota strains isolated from soil [85], however only one of four Acidobacteriota MAGs contained both types of cytochrome oxidases, but this might be due to incomplete assembly. Overall, this reflects that bacteria colonizing the gray fractures have adapted to the conditions with the ability to thrive under fluctuating oxygen concentrations.

The genetic potential to degrade carbohydrates varied largely among MAGs. The ability to degrade e.g. xylan, peptidoglycan, chitin and beta glucan suggests a broad appetite for carbohydrates coming from plants, bacteria, fungi and soil microfauna. *Kribbella* (MAG_F06), one of the enriched taxa in the gray fractures, contained the highest number of CAZymes of the MAGs identified. In support of this, *Kribbella* strains grow on a wide range of organic compounds as sole carbon and energy sources [77]. Among these is oxalic acid, released from plants into soil when they die [77], suggesting that decaying plants are an important source of organic carbon for this genus. The high abundance of chitin degrading enzymes found in the MAG suggests that remnants of fungi, earthworms and nematodes are important carbon sources. Earthworms were detected in the biopores at 120 cm bgs at the site [11] and the findings of the obligate nematode-symbionts Candidatus Xiphinematobacter [86] at 150 cm bgs indicate that nematodes are present in the gray fractures. Finally, the higher abundance of fungi in preferential flow paths at 150 cm and 75 cm compared to the matrix further supports this. The CAZyme profile of the enriched Candidatus Xiphenematobacter from Chtoniobacteriales (MAGs 13 and 18) showed a high proportion of mannanases and beta-glucanases suggesting their potential to degrade plant material [87, 88].

Contrasting this, only MAG_F11 showed potential to fix CO_2_ through the Calvin-Benson cycle, while still containing multiple carbohydrate active enzymes, indicative of a mixotrophic lifestyle. This demonstrates that heterotrophy is much more common than autotrophy in the gray fractures. Among the MAGs, the potential for anaerobic cobalamin biosynthesis dominated over aerobic pathways. We found near-complete anaerobic cobalamin biosynthesis pathways in MAGs belonging to Verrucomicrobiota, which have not been found previously to our knowledge. Further, the enriched genus *Rhodoplanes* from Rhizobiales (MAG_F11 (Proteobacteria)) also had the ability to biosynthesize cobalamin anaerobically. While it is energetically expensive to produce cobalamin, the potential to produce it also makes a strain more independent of other taxa. The ecological implications of this production could as a minimum be two-folded, although they are not mutually exclusive. Firstly, it could be an advantage in early colonization of a new environment, as suggested by [89]. This could in turn explain the higher abundance of the two enriched genera in the gray fractures, and the higher abundance of the phylum Verrucomicrobiota in the biopores, as rain fall could remove part of the microbial communities and opens new patches of soil for colonization. Secondly, these genera could provide cobalamin to other members of the community in return for other necessary organic compounds as observed between bacteria and algae in the marine environment [90, 91]. In fact, soil Verrucomicrobiota have multiple vitamin and amino acid auxotrophies [92]. In support of this, most of the recovered MAGS encode for one or several genes where cobalamin is needed as a co-factor (*mutA*,* metH*, and *rsmB*) [89, 93], indicating that cobalamin providers are needed in the community. The missing genes in three MAGs could be explained by lower completeness in these genomes, especially since MAG_F38 from the Anaerolineae is from the same order as MAG16 that contained two of the searched genes.

Providers of cobalamin in the ocean have, like the symbiont *Xiphinematobacter*, also been found to live in close proximity to other organisms [94]. Verrucomicrobiota correlated with dissolved organic carbon in the shallow subsurface [66] and have also been found in high abundance in rhizosphere [95]. Taken together, the ability to metabolize plant-derived C and N, in combination with biosynthesis of essential cobalamin can explain the increased relative abundance of *Xiphinematobacter*, *Rhodoplanes* and *Kribella* in preferential flow paths compared to matrix sediments.

## Conclusion

Here, we provide insight of the microbial ecology in preferential flow paths from typical subsurface clayey till below an agricultural field. Contrasting our first hypothesis, preferential flow paths had no effects on fungal communities. Despite this, there was a higher fungal abundance in preferential flow paths. We found bacterial and archaeal communities to be affected by preferential flow paths, demonstrating that factors governing the composition of these communities differ from fungi.

The seasonal variations of the water table resulting in varying oxygen conditions over the year was reflected in the genomic potential of the MAGs, in support of our second hypothesis. Further, the enriched *Kribella* showed potential for use a wide range of organic carbon source reflecting a highly versatile lifestyle adapted for the perceived varying carbon input in the gray fractures. This and the general trend of heterotrophic lifestyles of MAGs suggest that plant-derived material is an important source of carbon also supporting our second hypothesis.

We propose that future studies should include the activity of the microbial communities in the preferential flow paths before and after rain events and as a response to seasonal water table fluctuations. In addition, sampling should be expanded to elucidate spatiotemporal variability in general. This will, in particular when linked to determination of the connectivity to topsoil and taking the high prevalence of fractures into account, improve our understanding of the microbial contribution to geochemical cycling and release of greenhouse gases.

### Electronic supplementary material

Below is the link to the electronic supplementary material.

Supplementary Material 1

Supplementary Material 2

Supplementary Material 3

Supplementary Material 4

Supplementary Material 5

## Data Availability

Sequence data generated during this study are deposited in the NCBI BioProject Database with the BioProject accession number PRJNA606678. An overview of BioSample accession numbers can be found in Tables S1, S3-4.
