# Supplementary material for: Microbial life in preferential flow paths in subsurface clayey till revealed by metataxonomy and metagenomics
Source: BMC Microbiol. 2024 Aug 9;24:296. doi: 10.1186/s12866-024-03432-z (PMC11312239; doi:10.1186/s12866-024-03432-z)
Supplement: Supplementary file 1 — Supplementary Material 1 [file 12866_2024_3432_MOESM1_ESM.docx]

Microbial life in preferential flow paths in subsurface clayey till revealed by metataxonomy and metagenomics

Frederik Bak^1^, Christoph Keuschnig^2^, Ole Nybroe^1^, Jens Aamand^3^, Peter R. Jørgensen^4^, Mette H. Nicolaisen^1^, Timothy M. Vogel^5^, Catherine Larose^5^

^1^University of Copenhagen, Department of Plant and Environmental Sciences, Frederiksberg, Denmark, ^2^Université de Lyon, École Centrale de Lyon, CNRS, Laboratoire Ampere, Ecully, France, ^3^Geological Survey of Denmark and Greenland, Copenhagen, Denmark,  ^4^M E C ApS, Farum, Denmark, ^5^Universite Claude Bernard Lyon 1, Laboratoire d’Ecologie Microbioenne, UMR CNRS 5557, UMR INRAE 1418, VetAgro Sup, Villeurbanne, France, ^6^IGE – Institut de Géosciences de l’Environnement, Grenoble, France

**Supplementary figures and tables**

|  | Page |
| --- | --- |
| Fig. S1. Pictures from the excavation | 2 |
| Fig. S2. Rarefaction curves 16S rRNA gene reads | 3 |
| Fig. S3. Rarefaction curves ITS region 2 reads | 4 |
| Fig. S4. Relative abundances of five most abundant fungal phyla | 5 |
| Fig. S5. Relative abundances of 16 most abundant fungal orders | 6 |
| Fig. S6. NMDS ordinations of 16S rRNA amplicons at each depth | 7 |
| Fig. S7. Relative abundance of most abundant bacterial phyla | 8 |
| Fig. S8. Relative abundance of archaea | 8 |
| Fig. S9. Relative abundance of 10 most abundance bacterial and archaeal families | 9 |
| Fig. S10. Differentially abundant genera at 150 cm depth | 10 |
| Fig. S11. Abundance of CAZymes in each MAG | 11 |
| Table S2. Primer Sequences | 11 |
| Table S6. PERMANOVA results for ITS region 2 | 12 |
| Table S7. PERMANOVA results for 16S rRNA | 13 |
| References | 14 |

**Figure S1.** Pictures from the excavation. Above) The picture shows exposed grey fracture surfaces with rapid Brilliant Blue transport along a dense system of root macropores located inside the fractures. Dye tracer penetration from 1 to 2 m bgs occurred in less than 1 hour under natural infiltration. Adapted from (Jørgensen et al. 2022). Below) The picture shows exposed red fracture surfaces with dye tracer penetration of less than 10 cm after 42 days under forced infiltration with Brilliant Blue tracer dye.

| 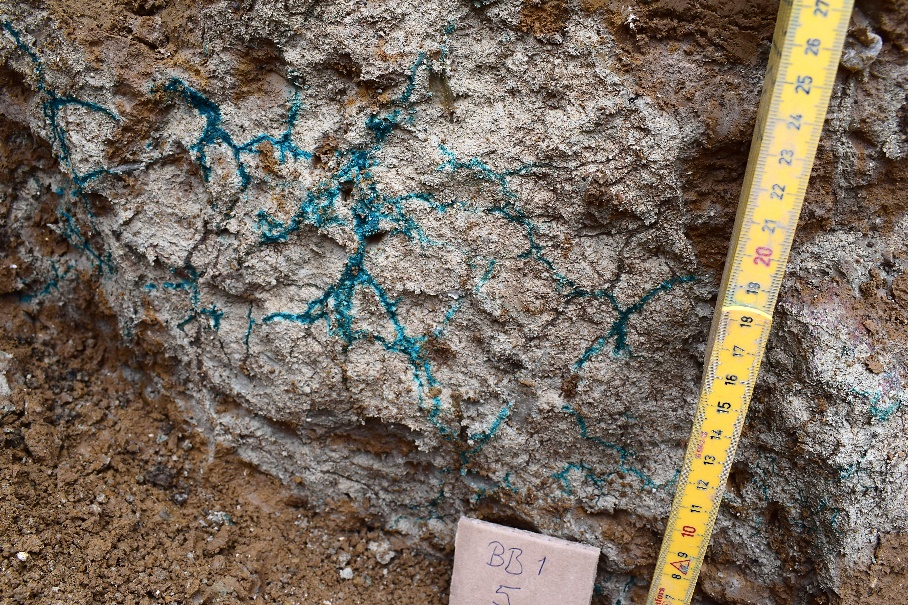 |
| --- |
| 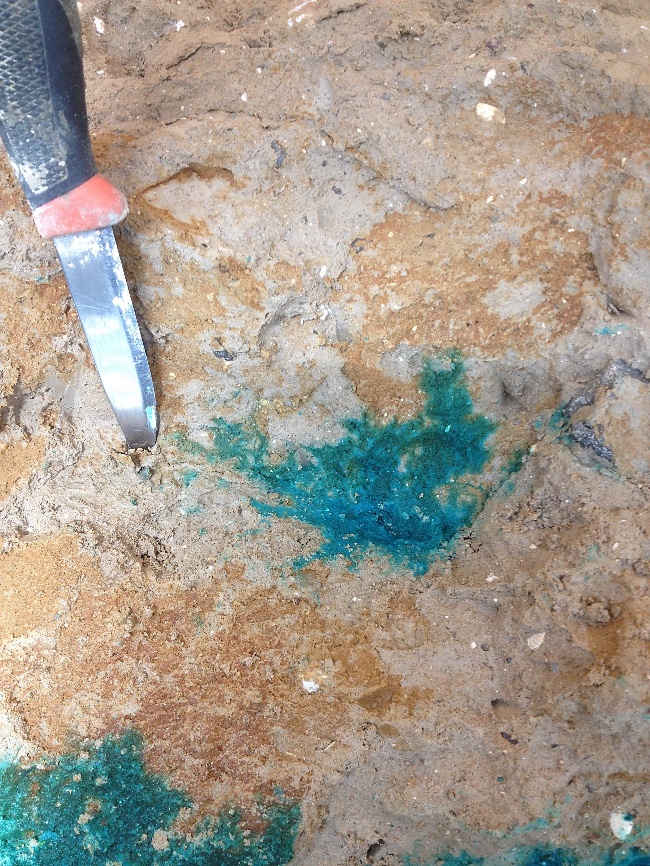 |

**Figure S2**. Rarefaction curves of 16S rRNA gene reads for the different niches. Sampling depth (cm below ground surface) is shown in the title of each plot.


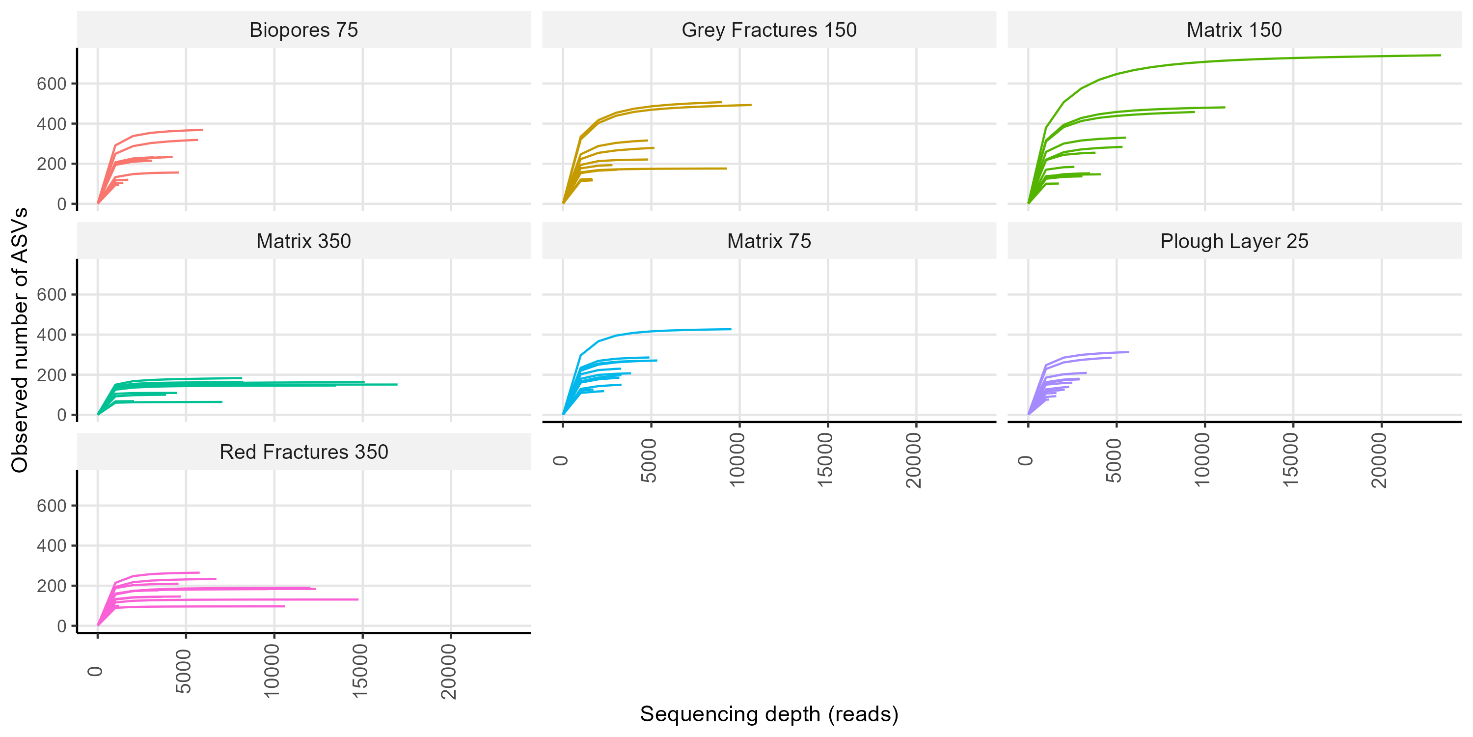


**Figure S3**. Rarefaction curves of ITS2 reads for the different niches. Sampling depth (cm below ground surface) is shown in the title of each plot.


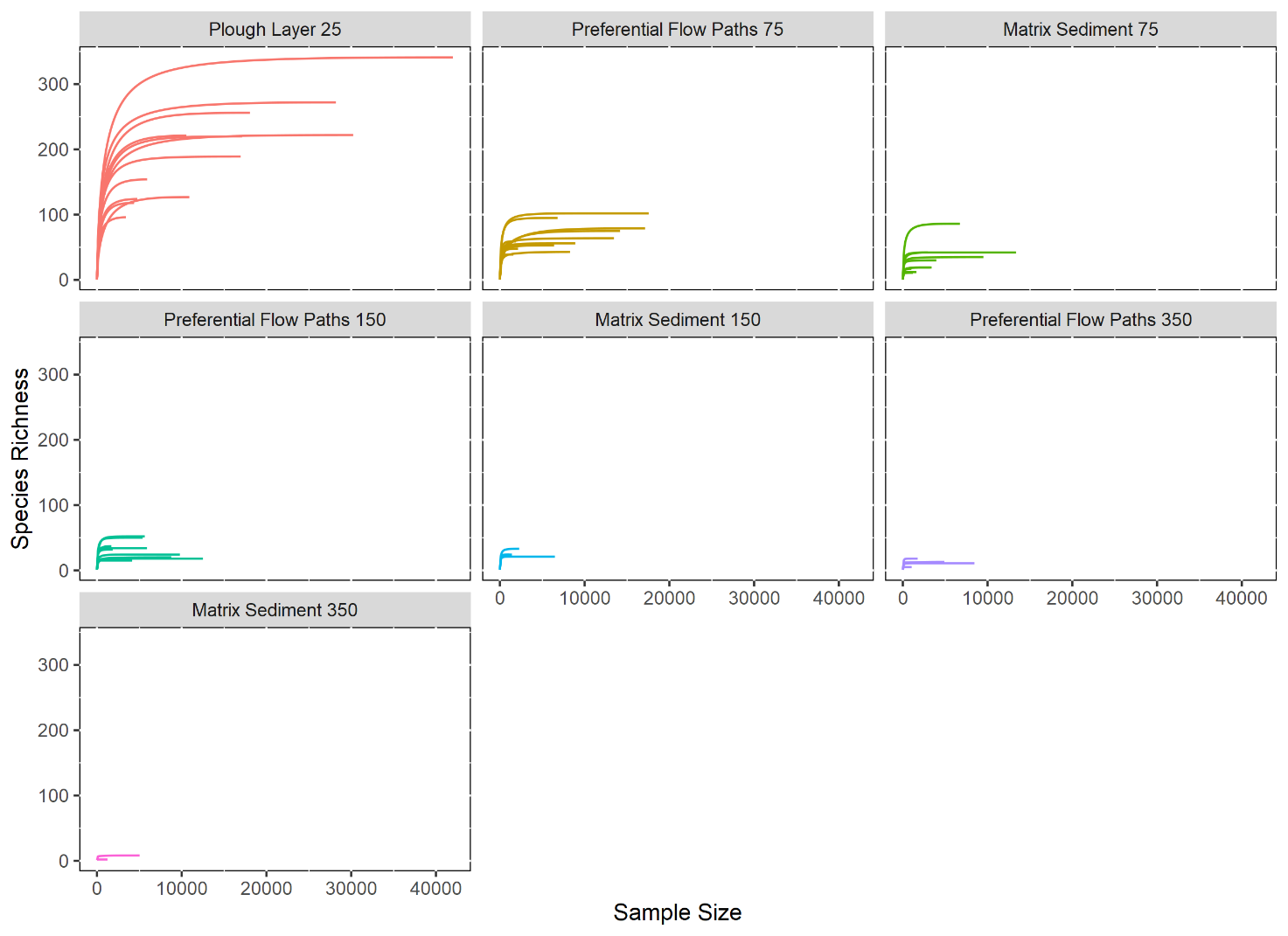


**Figure S4.** The five most abundant fungal phyla across all depths. Sampling depths (25, 75, 150 and 350 cm depth) are indicated on the y-axis.


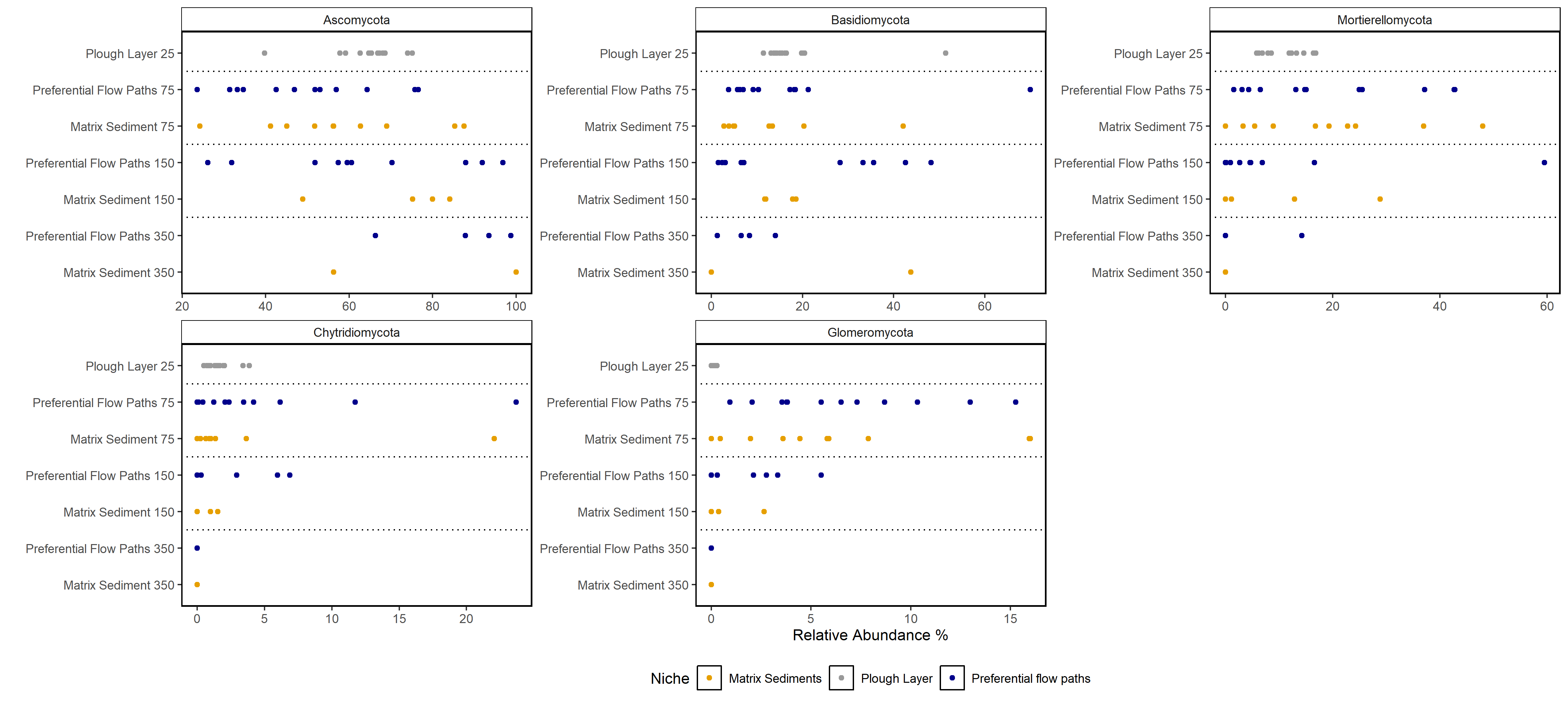


**Figure S5**. Relative abundance of 12 most abundant fungal orders across domains. Each point represents an individual sample. The dashed lines separate the different sampling depths in the profile). Sampling depths (25, 75, 150 and 350) are shown as cm below ground surface.


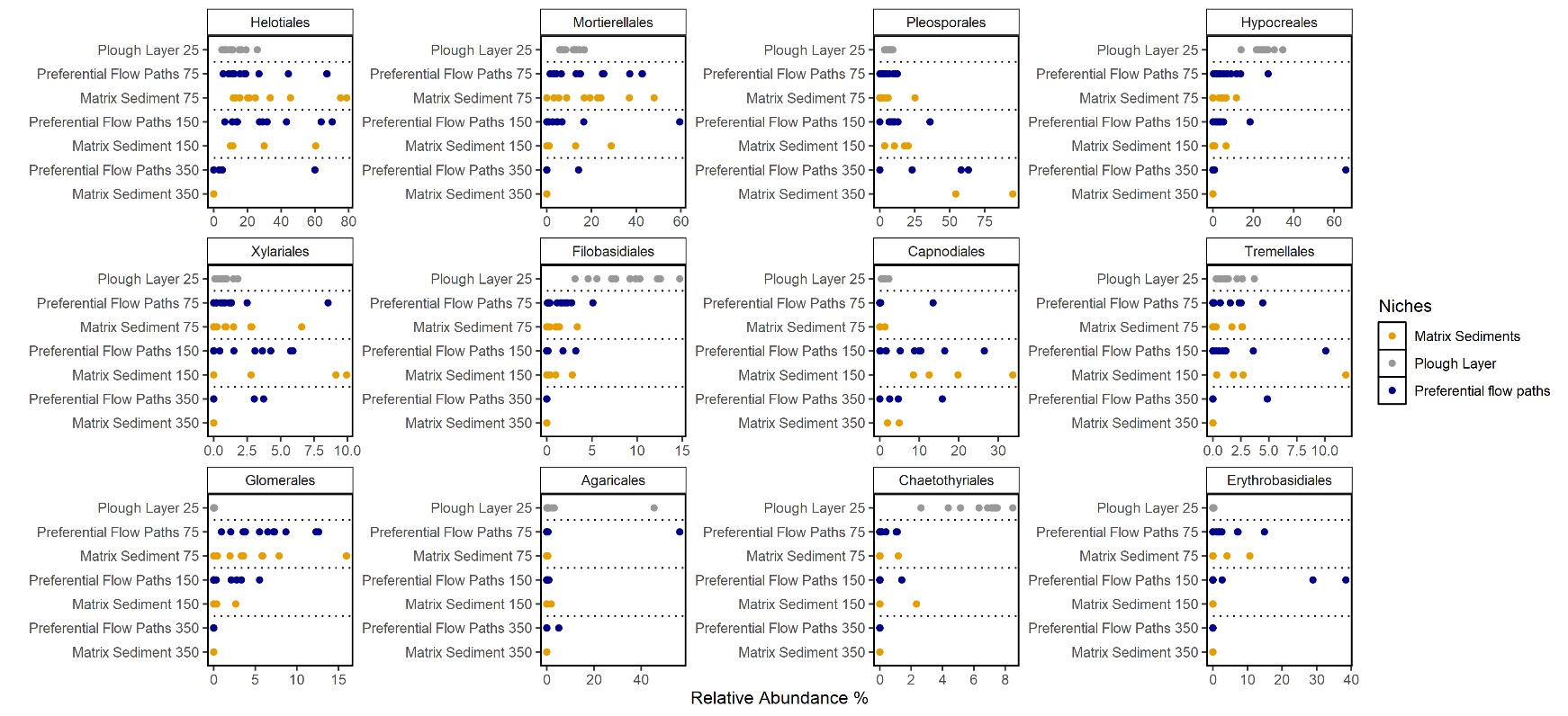


**Figure S6**. NMDS ordinations of Bray-Curtis dissimilarities between bacterial/archaeal communities based on the V4 region of the 16S rRNA gene at 75 cm (A), 150 cm (B), and 350 cm depth (C).


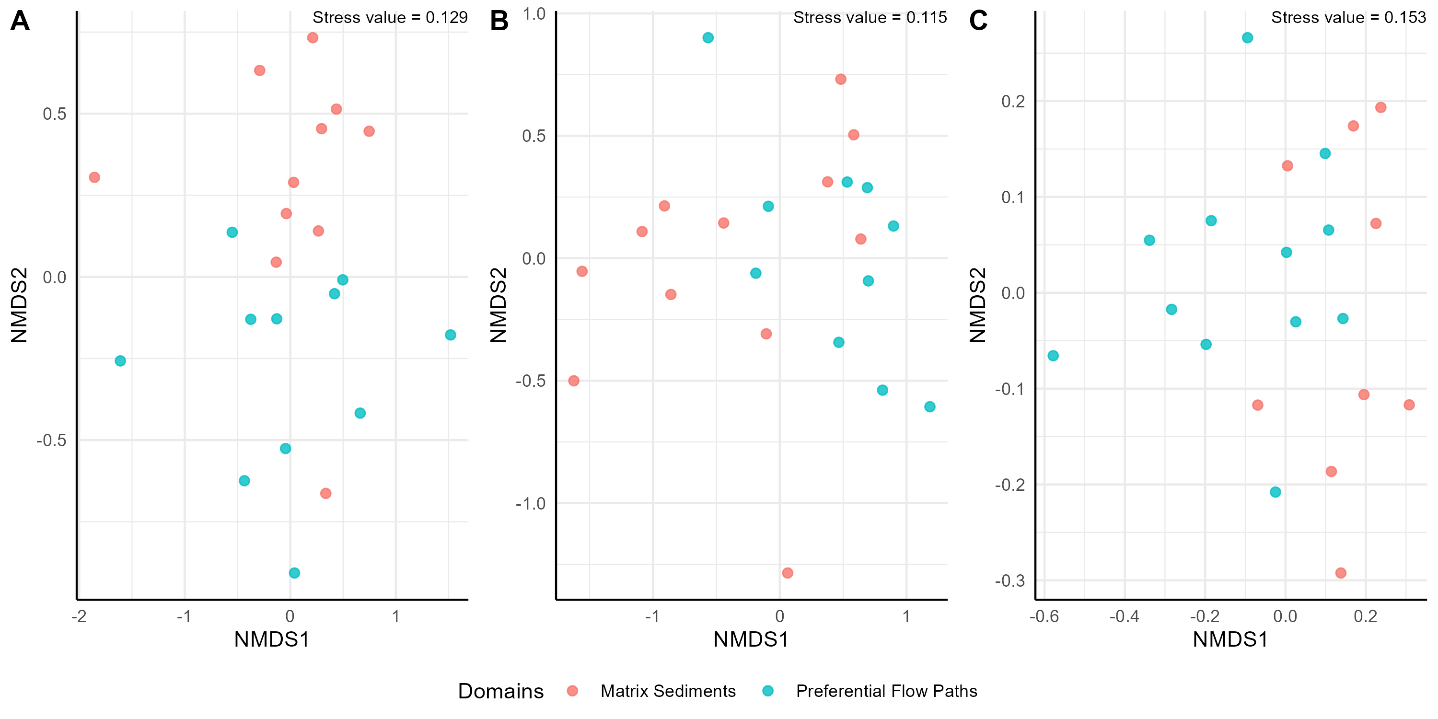


**Figure S7.** The 15 most abundant bacterial and archaeal phyla across all depths. Sampling depths (25, 75, 150 and 350 cm depth) are indicated on the y-axis. Phyla are depicted in alphabetical order.


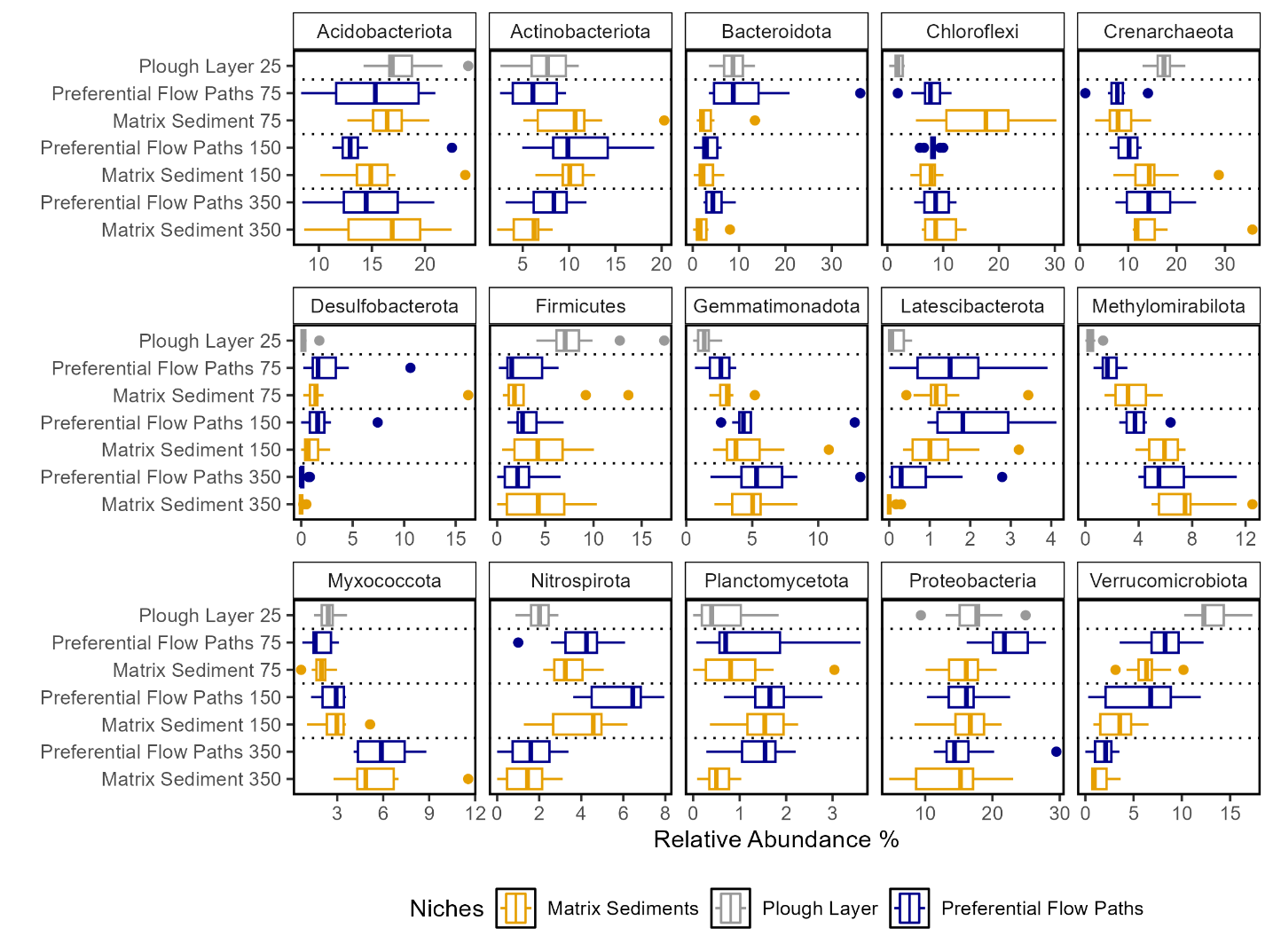


**Figure S8.** Relative abundance of bacteria and archaea (top) and Archaeal phyla (bottom) out of the total number of 16S rRNA sequences.


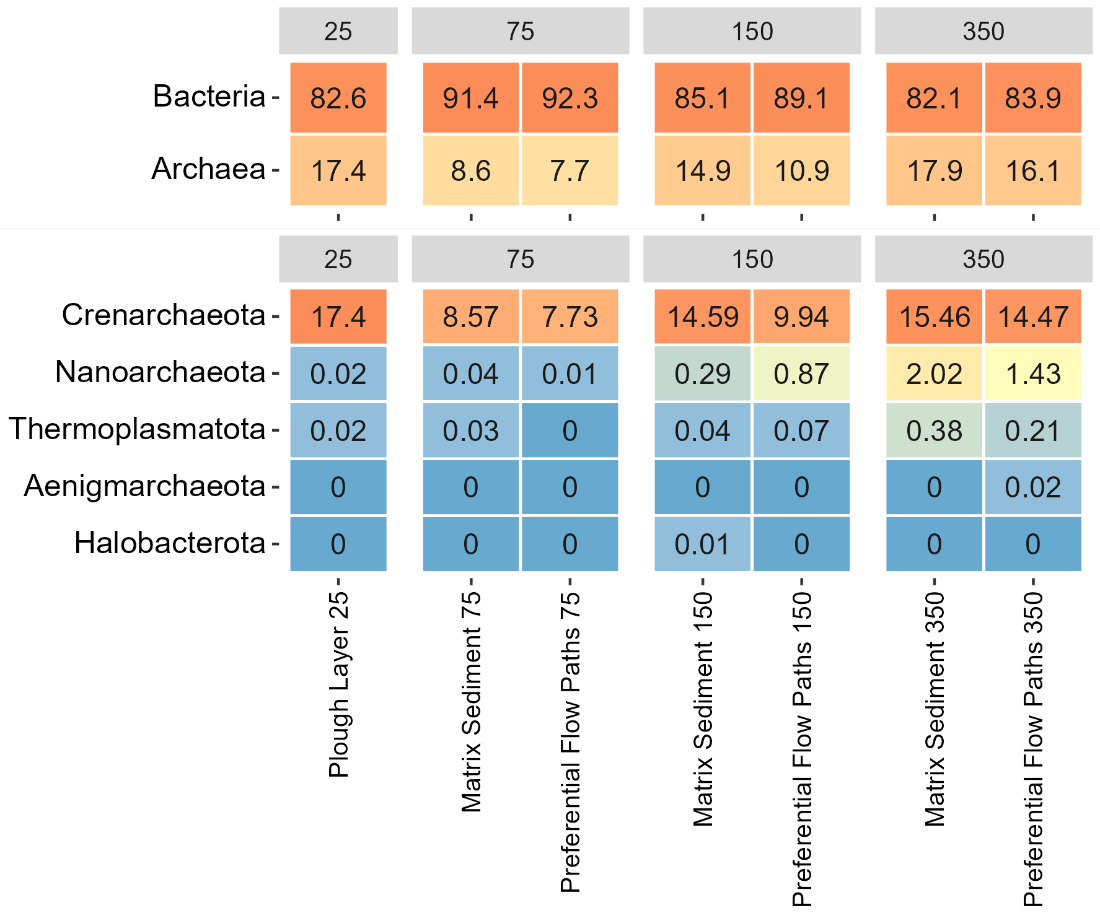


**Figure S9.** Relative abundance of the 10 most abundant bacterial and archaeal families across depths.


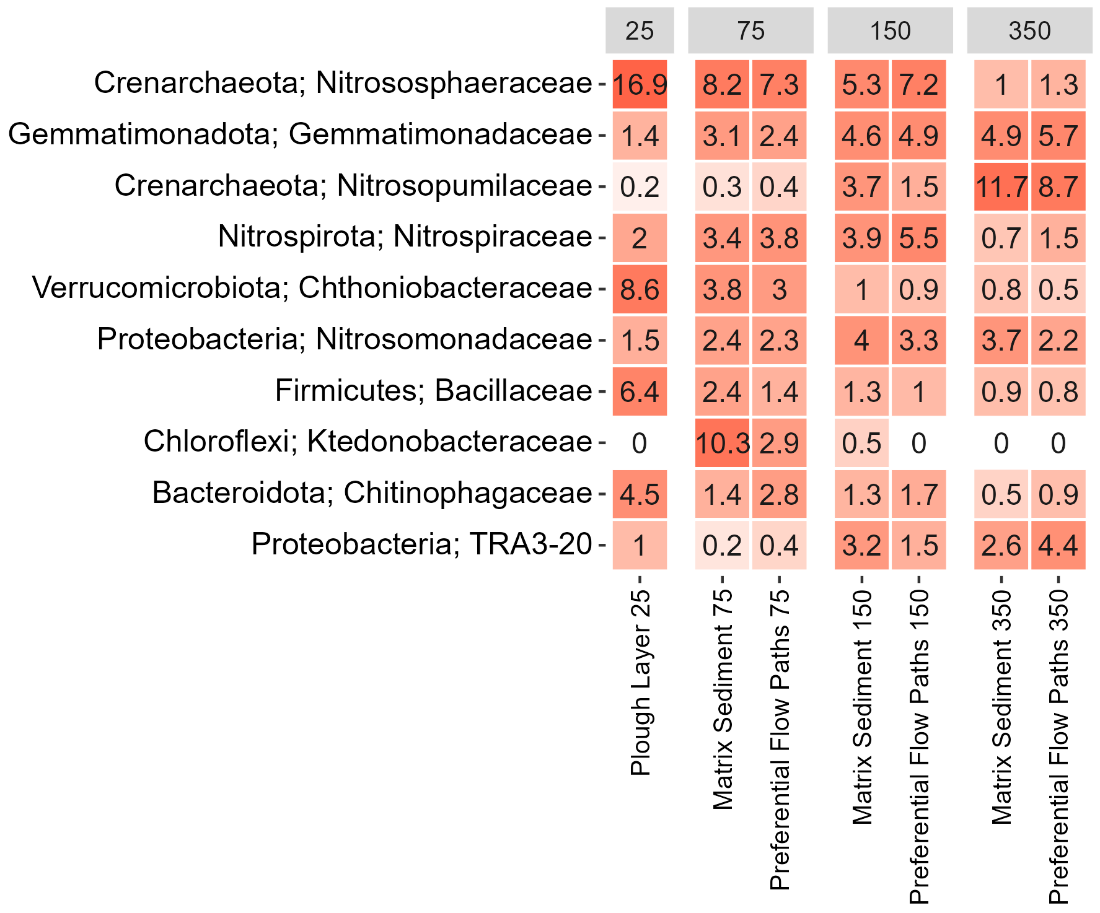


**Figure S10**. Differential abundance of bacterial genera at 150 cm. Genera at the left side of the punctuated line are more abundant in the preferential flow paths, and genera to the right side are more abundant in the matrix sediment (corncob, q < 0.05).

**
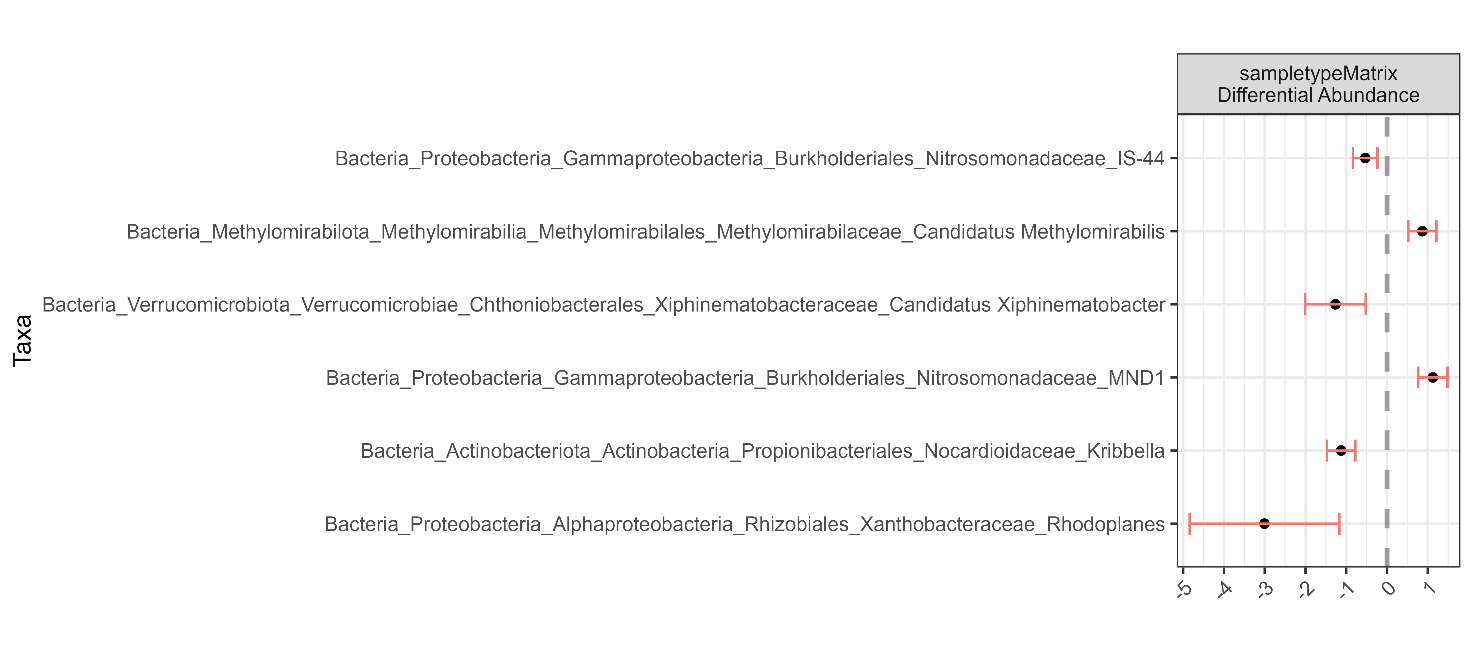
**

**Figure S11.** Abundance of CAZymes profiled using DIAMOND blast in each MAG.


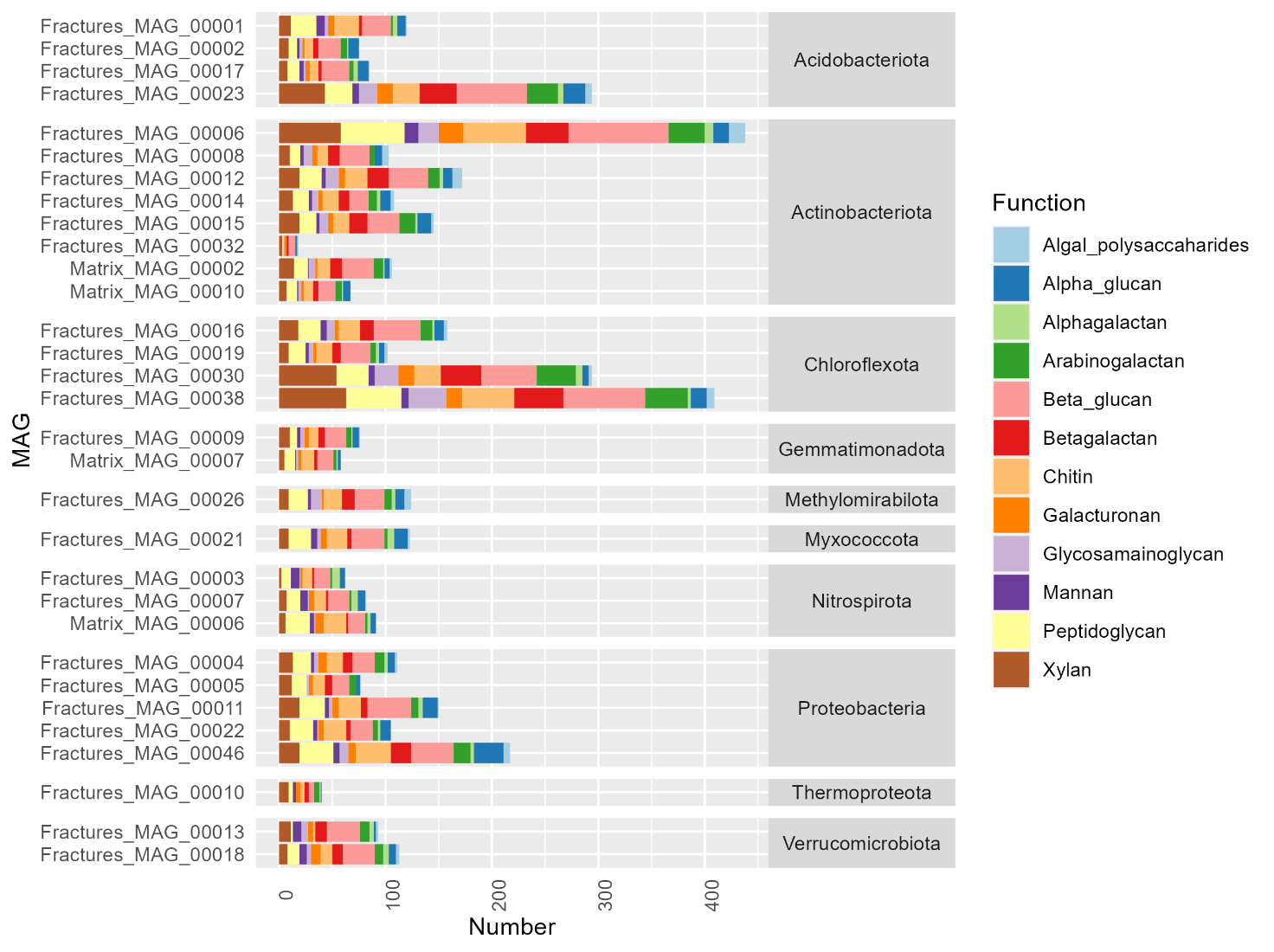


| Table S2. Primers used in this study. | | |
| --- | --- | --- |
| Primer | Sequence | Reference |
| **16S Illumina** |  |  |
| 515F-Y | GTGYCAGCMGCCGCGGTAA | (Parada, Needham, and Fuhrman 2016) |
| 806R | GGACTACHVGGGTWTCTAAT | (Caporaso et al. 2011) |
|  |  |  |
| **16S qPCR** |  |  |
| 1369F | CGGTGAATACGTTCYCGG | (Suzuki, Taylor, and DeLong 2000) |
| 1492R | GGWTACCTTGTTACGACTT | (Suzuki, Taylor, and DeLong 2000) |
| TM1389F | CTTGTACACACCGCCCGTC | (Suzuki, Taylor, and DeLong 2000) |
|  |  |  |
| **ITS2 Illumina** | |  |
| 5.8S-FUN | AACTT TYRRCAAYGGATCWCT | (Taylor et al. 2016) |
| ITS4-FUN | AGCCTCCGCTTA TTGATATGCTTAART | (Taylor et al. 2016) |
|  |  |  |
| **ITS1 qPCR** |  |  |
| ITS1-F | CTTGGTCATTTAGAGGAAGTAA | (Gardes and Bruns 1993) |
| ITS2 | GCTGCGTTCTTCATCGATGC | (White et al. 1990) |

Table S6. Results of PERMANOVA analysis for the three depths below the plough layer for ITS2 region.

| **Factor** | **DF** | **SumsOfSqs** | **F.model** | **R^2^** | **Pr(>F)** |
| --- | --- | --- | --- | --- | --- |
| Depth | 2 | 2.9026 | 3.7695 | 0.1609 | 0.001 |
| Niche | 1 | 0.4758 | 1.2358 | 0.0264 | 0.139 |
| Interaction | 2 | 0.7973 | 1.0354 | 0.0442 | 0.367 |
| Residuals | 36 | 13.8602 |  | 0.7685 |  |
| Total | 41 | 18.0358 |  | 1.0000 |  |

Table S7. Results of PERMANOVA analysis for the three depths below the plough layer for the 16S rRNA gene together (top panel), and each depth individually (lower panels).

| **Factor** | | **DF** | | **SumsOfSqs** | | **F.model** | | **R^2^** | | **Pr(>F)** | |
| --- | --- | --- | --- | --- | --- | --- | --- | --- | --- | --- | --- |
| Depth | | 2 | | 5.0870 | | 7.6206 | | 0.1913 | | 0.001 | |
| Niche | | 1 | | 0.6102 | | 1.8283 | | 0.02295 | | 0.018 | |
| Interaction | | 2 | | 1.2032 | | 1.8024 | | 0.04524 | | 0.001 | |
| Residuals | | 59 | | 19.692 | |  | | 0.7405 | |  | |
| Total | | 64 | | 26.593 | |  | | 1.0000 | |  | |
| PERMANOVA results, samples at 75 cm depth, Controlling for pair like structure of the data (by = “margin”) | | | | | | | | | | | |
| **Factor** | | **DF** | | **SumsOfSqs** | | **F.model** | | **R^2^** | | **Pr(>F)** | |
| Pair | | 11 | | 4.1133 | | 1.5104 | | 0.5963 | | 0.001 | |
| Niche | | 1 | | 0.5424 | | 2.1910 | | 0.0786 | | 0.001 | |
| Residuals | | 9 | | 2.2281 | |  | | 0.3230 | |  | |
| Total | | 21 | | 6.8985 | |  | | 1.0000 | |  | |
| PERMANOVA results, samples at 150 cm depth, Controlling for pair like structure of the data (by = “margin”) | | | | | | | | | | | |
| **Factor** | | **DF** | | **SumsOfSqs** | | **F.model** | | **R^2^** | | **Pr(>F)** | |
| Pair | | 10 | | 3.7329 | | 1.2476 | | 0.5054 | | 0.027 | |
| Niche | | 1 | | 0.6206 | | 2.0741 | | 0.0840 | | 0.003 | |
| Residuals | | 10 | | 2.9921 | |  | | 0.4051 | |  | |
| Total | | 21 | | 7.3867 | |  | | 1.000 | |  | |
| PERMANOVA results, samples at 350 cm depth, Controlling for pair like structure of the data (by = “margin”) | | | | | | | | | | |  |
| **Factor** | **DF** | | **SumsOfSqs** | | **F.model** | | **R^2^** | | **Pr(>F)** | |  |
| Pair | 11 | | 4.2129 | | 1.2698 | | 0.5835 | | 0.003 | |  |
| Niche | 1 | | 0.4779 | | 1.5845 | | 0.0662 | | 0.008 | |  |
| Residuals | 8 | | 2.4129 | |  | | 0.3342 | |  | |  |
| Total | 20 | | 7.2205 | |  | | 1.0000 | |  | |  |

**References**

Caporaso, J. Gregory, Christian L. Lauber, William A. Walters, Donna Berg-Lyons, Catherine A. Lozupone, Peter J. Turnbaugh, Noah Fierer, and Rob Knight. 2011. “Global Patterns of 16S rRNA Diversity at a Depth of Millions of Sequences per Sample.” *Proceedings of the National Academy of Sciences of the United States of America* 108 (SUPPL. 1): 4516–22. https://doi.org/10.1073/pnas.1000080107.

Gardes, M., and T. D. Bruns. 1993. “ITS Primers with Enhanced Specificity for Basidiomycetes ‐ Application to the Identification of Mycorrhizae and Rusts.” *Molecular Ecology* 2 (2): 113–18. https://doi.org/10.1111/j.1365-294X.1993.tb00005.x.

Jørgensen, Peter R., Paul Henning Krogh, Jiayi Qin, Luca Modesti, Ida Broman Nielsen, Frederik Seersholm, Natascha Wagner, et al. 2022. “Ancient Root Macropores and Fractures in Glacial till and Their Contribution to Pesticide Vulnerability of Groundwater in Low and High-Ground Agricultural Landscape PESTPORE2.” 207. Pesticide Research. The Danish Environmental Protection Agency. https://mst.dk/service/publikationer/publikationsarkiv/2022/feb/pestpore2/.

Parada, Alma E., David M. Needham, and Jed A. Fuhrman. 2016. “Every Base Matters: Assessing Small Subunit rRNA Primers for Marine Microbiomes with Mock Communities, Time Series and Global Field Samples.” *Environmental Microbiology* 18 (5): 1403–14. https://doi.org/10.1111/1462-2920.13023.

Suzuki, M T, L T Taylor, and E F DeLong. 2000. “Quantitative Analysis of Small-Subunit rRNA Genes in Mixed Microbial Populations via 5’-Nuclease Assays.” *Applied and Environmental Microbiology* 66 (11): 4605–14.

Taylor, D. Lee, William A. Walters, Niall J. Lennon, James Bochicchio, Andrew Krohn, J. Gregory Caporaso, and Taina Pennanen. 2016. “Accurate Estimation of Fungal Diversity and Abundance through Improved Lineage-Specific Primers Optimized for Illumina Amplicon Sequencing.” *Applied and Environmental Microbiology* 82 (24): 7217–26. https://doi.org/10.1128/AEM.02576-16.

White, T.J., T. Bruns, S. Lee, and J. Taylor. 1990. “AMPLIFICATION AND DIRECT SEQUENCING OF FUNGAL RIBOSOMAL RNA GENES FOR PHYLOGENETICS.” *PCR Protocols*, January, 315–22. https://doi.org/10.1016/B978-0-12-372180-8.50042-1.
